# Functional molecule-mediated assembled copper nanozymes for diabetic wound healing

**DOI:** 10.1186/s12951-023-02048-1

**Published:** 2023-08-25

**Authors:** Wenyan Huang, Ping Xu, Xiaoxue Fu, Jiaxin Yang, Weihong Jing, Yucen Cai, Yingjuan Zhou, Rui Tao, Zhangyou Yang

**Affiliations:** 1 Chongqing Key Laboratory for Pharmaceutical Metabolism Research, Chongqing Pharmacodynamic Evaluation Engineering Technology Research Center, College of Pharmacy, Chongqing Medical University, Chongqing, 400016 China; 2 Department of Hepatobiliary Surgery, Bishan hospital, Chongqing Medical University, Chongqing, 402760 China

**Keywords:** Diabetic wound healing, Nanozymes, Multicatalytic activity, Angiogenesis effect, Photothermal response

## Abstract

**Background:**

The complex hyperglycemic, hypoxic, and reactive oxygen species microenvironment of diabetic wound leads to vascular defects and bacterial growth and current treatment options are relatively limited by their poor efficacy.

**Results:**

Herein, a functional molecule-mediated copper ions co-assembled strategy was constructed for collaborative treatment of diabetic wounds. Firstly, a functional small molecule 2,5-dimercaptoterephthalic acid (DCA) which has symmetrical carboxyl and sulfhydryl structure, was selected for the first time to assisted co-assembly of copper ions to produce multifunctional nanozymes (Cu-DCA NZs). Secondly, the Cu-DCA NZs have excellent multicatalytic activity, and photothermal response under 808 nm irradiation. In vitro and in vivo experiments showed that it not only could efficiently inhibit bacterial growth though photothermal therapy, but also could catalyze the conversion of intracellular hydrogen peroxide to oxygen which relieves wound hypoxia and improving inflammatory accumulation. More importantly, the slow release of copper ions could accelerate cellular proliferation, migration and angiogenesis, synergistically promote the healing of diabetic wound furtherly.

**Conclusions:**

The above results indicate that this multifunctional nanozymes Cu-DCA NZs may be a potential nanotherapeutic strategy for diabetic wound healing.

**Supplementary Information:**

The online version contains supplementary material available at 10.1186/s12951-023-02048-1.

## Background

Diabetes is a common chronic endocrine disease and the number of people affected is still on the rise [1–3]. Although the early acute hypoxic microenvironment due to inflammatory cell recruitment promotes cell proliferation [4–7], prolonged hypoxia in diabetic wound impairs the healing process via inhibition of angiogenesis, re-epithelialization [8]. Moreover, the failure of epithelial cell healing would exacerbate bacterial growth on the wound could lead to infection, aggravating wound deterioration [9, 10]. Therefore, enhanced wound angiogenesis is key to diabetic wound healing. Currently, conventional approach to the management of diabetic wound includes surgical debridement, dressing, infection control [11, 12], vascular assessment and glycaemic control. However, due to the complex microenvironment of diabetic wound, the prognosis of current therapies used to treat diabetic wound is not satisfactory. Thus, the efficient treatment strategies are essential to promote diabetic wound healing.

At present, some metal ions have attracted increasing research interest due to their vascularization effects [13, 14]. Especially for copper, as an essential trace element in human body [15], plays an important role in many enzymes, such as tyrosinase and Cu–Zn superoxide dismutase [16–18]. Besides, copper ions have been reported that could promote angiogenesis by inducing the production of vascular endothelial growth factor (VEGF) and thus accelerating wound healing [19–21]. However, copper ions alone have the disadvantage of sudden release as well as high local release concentrations, which could instead have side effects on the organism [22]. Recently, copper-based metal-organic framework (Cu-MOF) nanoparticles have been widely used in the study of chronic wound healing, which not only effectively promotes the healing of wound but also reduces the side effects of copper ions [22, 23]. However, the large and unstable particle sizes, and easily damaged structures of Cu-MOF making it difficult to ensure the controllability and reproducibility in the treatment process and lacks the regulation ability for the complex microenvironment of diabetic wound [24–26]. The above limitations require the development of a functional strategy to synergistically promote diabetic wound healing.

In recent years, nanozymes have attracted increasing interests from researchers due to their low cost, high stability, and catalytic activity [27, 28]. Especially for copper nanozymes, which have been used to treatment of oxidative stress and inflammatory diseases (for example parkinson’s, nephritis [29]) due to its unique and numerous enzyme activities. Although Cu-MOF has the potential to promote wound healing, it is difficult to obtain effective mimic enzyme activity due to its relatively large size and fragile structure. Therefore, there are few reports on diabetic wound repair based on copper nanozymes [30]. In addition, if we can give more functions to copper nanozymes while maintaining its intrinsic enzymatic activity, it may be more beneficial to the synergistic repair treatment of diabetic wound.

Inspired by the development of co-assembly technology [31], especially for small molecules coordinate with metal ions through their functional groups for constructing functional nanostructures which has potential biomedical application value. There are some studies found that the dual ligand combination of carboxyl and sulfhydryl groups provides a potentially powerful strategy for the formation of new ligand network structures [32, 33]. The potential advantages of this dual ligand binding come from the different characteristics of the carboxyl and sulfhydryl groups [34]: the ionic, chemically hard carboxyl group tends to bind the metal ion for network formation, while the softer sulfhydryl group could act as an independent secondary donor group. More importantly, small molecules containing sulfhydryl groups can act as functional assembly linkers and act as antioxidant drugs themselves at the same time [35]. Therefore, the development of a nanozyme based on the co-assembly of special small molecule containing sulfhydryl groups with copper ions will broaden the treatment of oxidative stress and inflammatory diseases, especially the treatment of diabetic wounds.

**Scheme 1:**
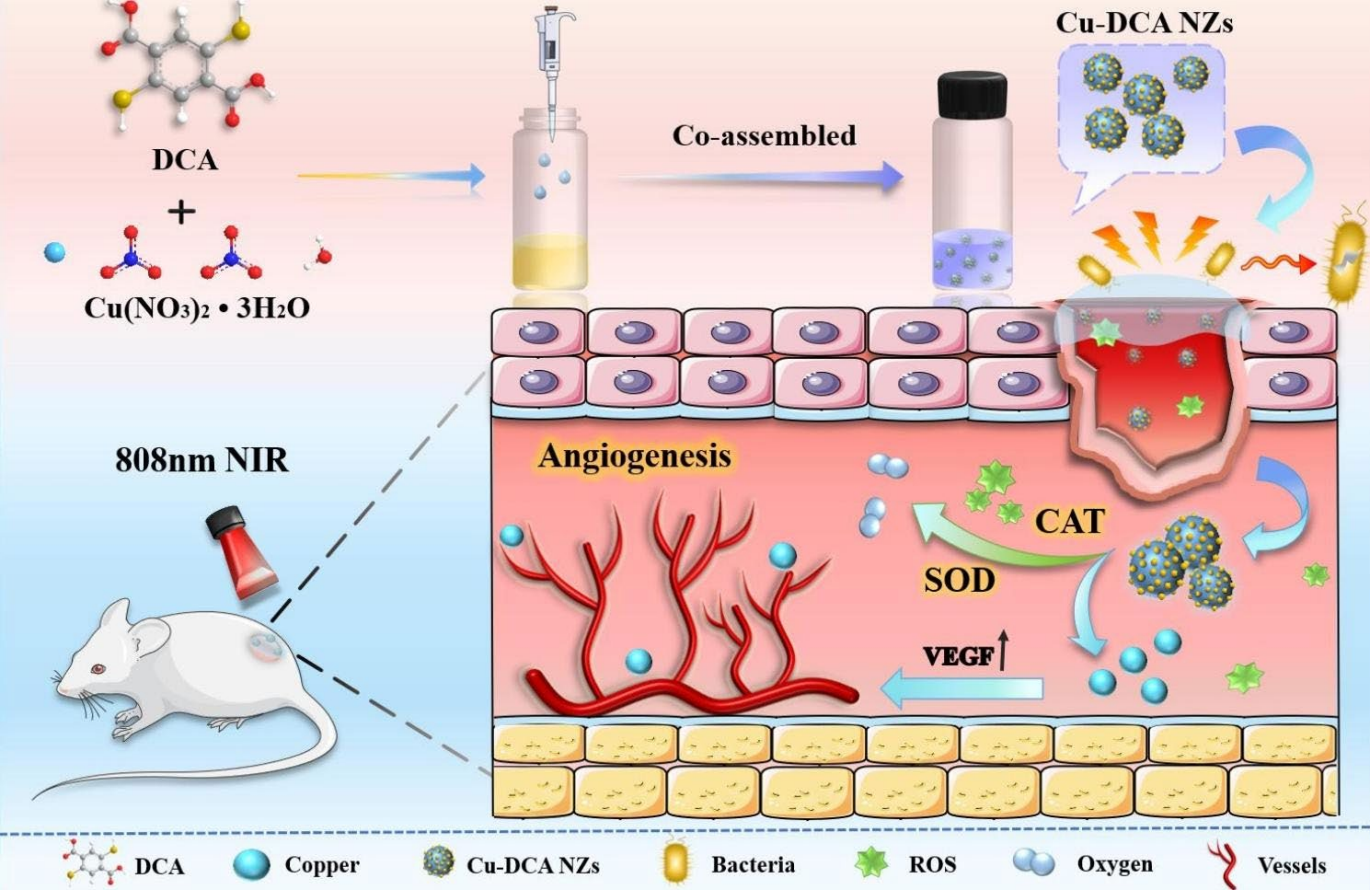
Schematic illustration of the preparation and application of Cu-DCA NZs for diabetic wound healing

Based on the above considerations, we designed a copper ions-mediated multifunctional nanozymes platform based on a co-assembly strategy for diabetic wound treatment. As shown in Scheme 1., a multifunctional nanozymes (Cu-DCA NZs) was prepared by simple and rapid co-assembly of a dual ligand molecule DCA which possess symmetric carboxyl and sulfhydryl groups with copper ions for the first time. The Cu-DCA NZs not only have the potential for drug delivery, but also could effectively inhibit bacterial growth by photothermal therapy under NIR irradiation. It also exerts efficient mimiced the activities of superoxide dismutase (SOD) and catalase (CAT) to catalyze the conversion of intracellular hydrogen peroxide (H_2_O_2_) to oxygen, thereby alleviating wound hypoxia and improving inflammation accumulation.

More importantly, the combination of copper ions accelerates cell proliferation, migration, and angiogenesis, further effectively promoting diabetic wound healing. In conclusion, Cu-DCA NZs accelerated the healing of diabetic wound effectively by synergistic therapeutic effects might shed light for future clinical translation on chronic wound reparation.

## Results

### Synthesis and characterization of Cu-DCA NZs

**Fig. 1:**
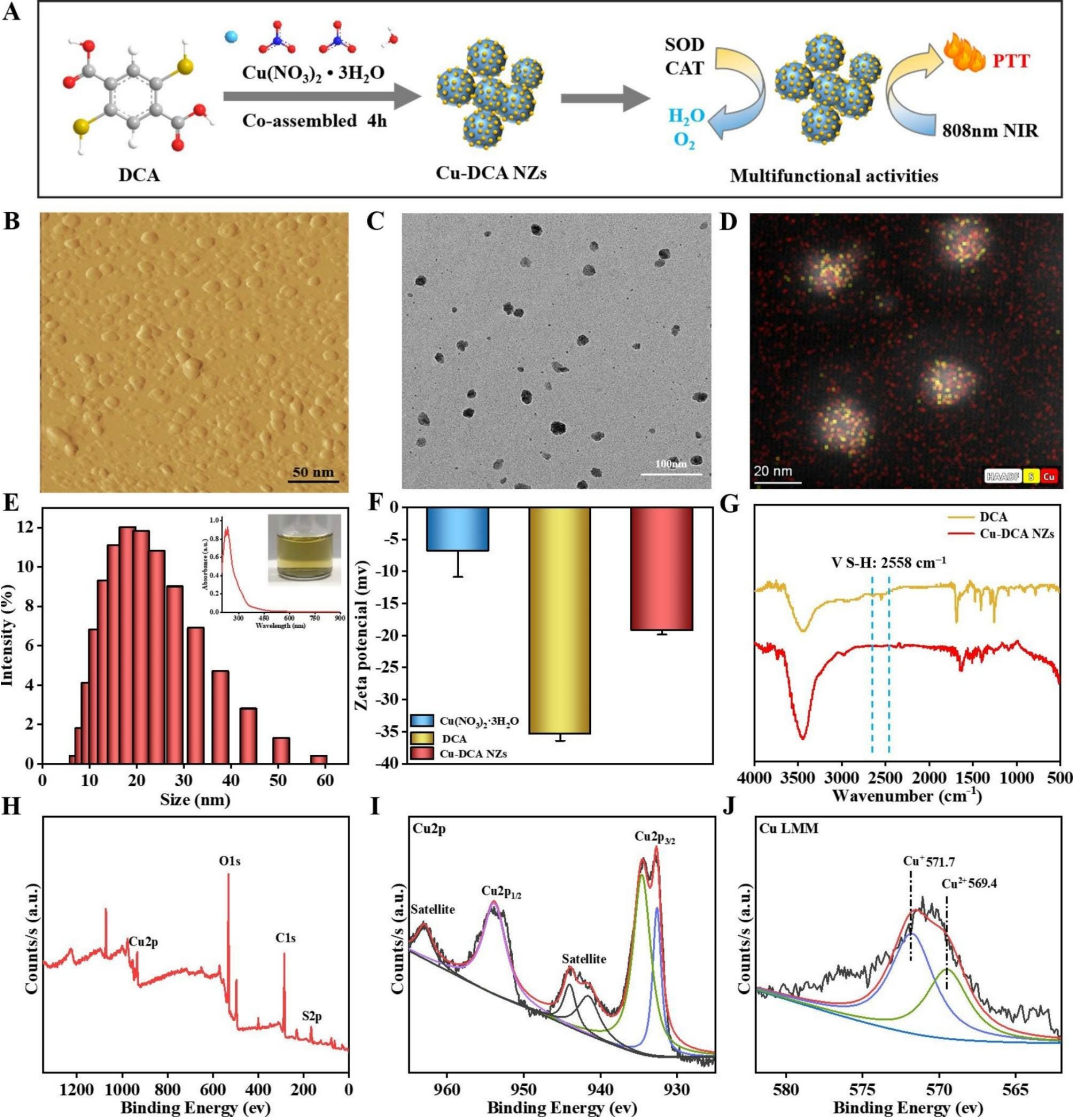
Characterization of Cu-DCA NZs. (**A**) Schematic of the preparation of Cu-DCA NZs. (**B**) AFM images of Cu-DCA NZs. (**C**) TEM images and (**D**) corresponding element mapping of Cu-DCA NZs. (**E**) The hydrated particle size of Cu-DCA NZs. Inset is the UV–vis absorption spectra and solution photograph of Cu-DCA NZs. (**F**) Zeta potential of Cu(NO_3_)_2_·3H_2_O, DCA and Cu-DCA NZs. (**G**) FTIR spectrum of DCA and Cu-DCA NZs. (**H**-**J**) XPS analysis of Cu-DCA NZs. (**H**) XPS spectra, (**I**) Cu 2p and (**J**) Cu LMM. Data are presented as the mean ± SD (n = 3)

The Cu-DCA NZs were synthesized by a green, simple and fast method (Fig. 1A). The Cu(NO_3_)_2_·3H_2_O solution was slowly added dropwise to the DCA solution under alkaline conditions and stirred for 4 h until the color of the solution changed from brownish black to a clear blue-purple. The AFM (Fig. 1B) and TEM images (Fig. 1C) showed that these Cu-DCA NZs were spherical particles with uniform dispersion of approximately 20.0–25.0 nm in the dry state. Then, the element distribution of Cu-DCA NZs was characterized by TEM in conjunction with energy-dispersive X-ray spectroscopy (TEM-EDS). As shown in the images of elemental mapping (Fig. 1D, S1), the distribution of sulfhydryl and copper preliminarily testified that Cu-DCA NZs were successfully synthesized. As shown in (Fig. 1E), the average hydrated particle size of Cu-DCA NZs were around 20.0 nm and no significant difference compared with AFM and TEM in particle size (polydisperse index 0.232). The physisorption experiments (Fig. S2) showed that the obtained Cu-DCA NZs displayed a Brunauer-Emmett-Teller (BET) surface area of 0.8500 m²/g and an average pore size of 4.3284 nm, which proved to be a mesoporous material and possessed potential drug loading capability. Moreover, the results of the zeta potential (Fig. 1F) indicated that the originally negatively charged DCA (− 35.3 mV) has an improved negative potential in Cu-DCA NZs (− 12.6 mV) obtained after reaction with copper ions (− 6.78 mV), further indicating the nanozymes were successfully synthesized. The DCA and copper ions bind way was initially verified by FTIR spectra (Fig. 1G). The band of approximately 2558 cm^− 1^ was observed in DCA, which is appointed as the existence of the v(S-H) vibration. However, the v(S-H) vibration disappeared in the Cu-DCA NZs, which could be caused by the coordination between sulfhydryl atoms in sulfhydryl groups and copper ions. In addition, the X-ray Powder diffractometer (XRD) pattern (Fig. S3) indicated that the Cu-DCA NZs were non-crystalline. Then, the original concentration of copper ions of Cu-DCA NZs was measured by ICP–MS to be 0.48mM (Table S1), and subsequent Cu-DCA NZs was quantified by copper ions concentration. Subsequently, to further understand the interactions between Cu-DCA NZs, XPS (Fig. 1H- J) had been used to investigate the surface properties and oxidation state of copper in nanozymes. The binding energy (BE) of the surface charge was corrected at 284.5 eV using the C1S peak of the contaminant carbon as a reference (Fig. 1H). The XPS spectra (Fig. 1I) of Cu2p showed BE peaks at ~ 932 eV and ~ 953 eV, corresponding to Cu2p3/2 and Cu2p1/2 respectively [36, 37]. In addition, Cu 2p3/2 has two contributions at ~ 934 and ~ 932 eV. The higher BE peak at ~ 934 eV is attributed to Cu^2+^ in Cu-DCA NZs accompanied by characteristic Cu^2+^ satellite peaks (938–948 eV) and 963 eV. The lower BE peak at ~ 932 eV suggests the presence of Cu^+^ or Cu^0^. However, the Cu2p spectra could not distinguish between Cu^+^ and Cu^0^, Auger Cu LMM spectra (Fig. 1J) were used to further confirm the presence of Cu^+^ at BE ~ 571 eV [38]. In summary, Cu-DCA NZs were confirmed to successfully synthesized, and the valence changes of copper ions may bring some potential biological activity.

### Photothermal performance of Cu-DCA NZs

The change of reaction color had attracted our interests and we speculated that Cu-DCA NZs may possess some photothermal effect, then the photothermal properties of Cu-DCA NZs was comprehensively evaluated. Normally, the molar ratio of reactants would influence the production of final nanoparticles. Therefore, we optimized reaction molar ratio to adjust the physiochemical properties and catalytic activity of Cu-DCA NZs.

**Fig. 2:**
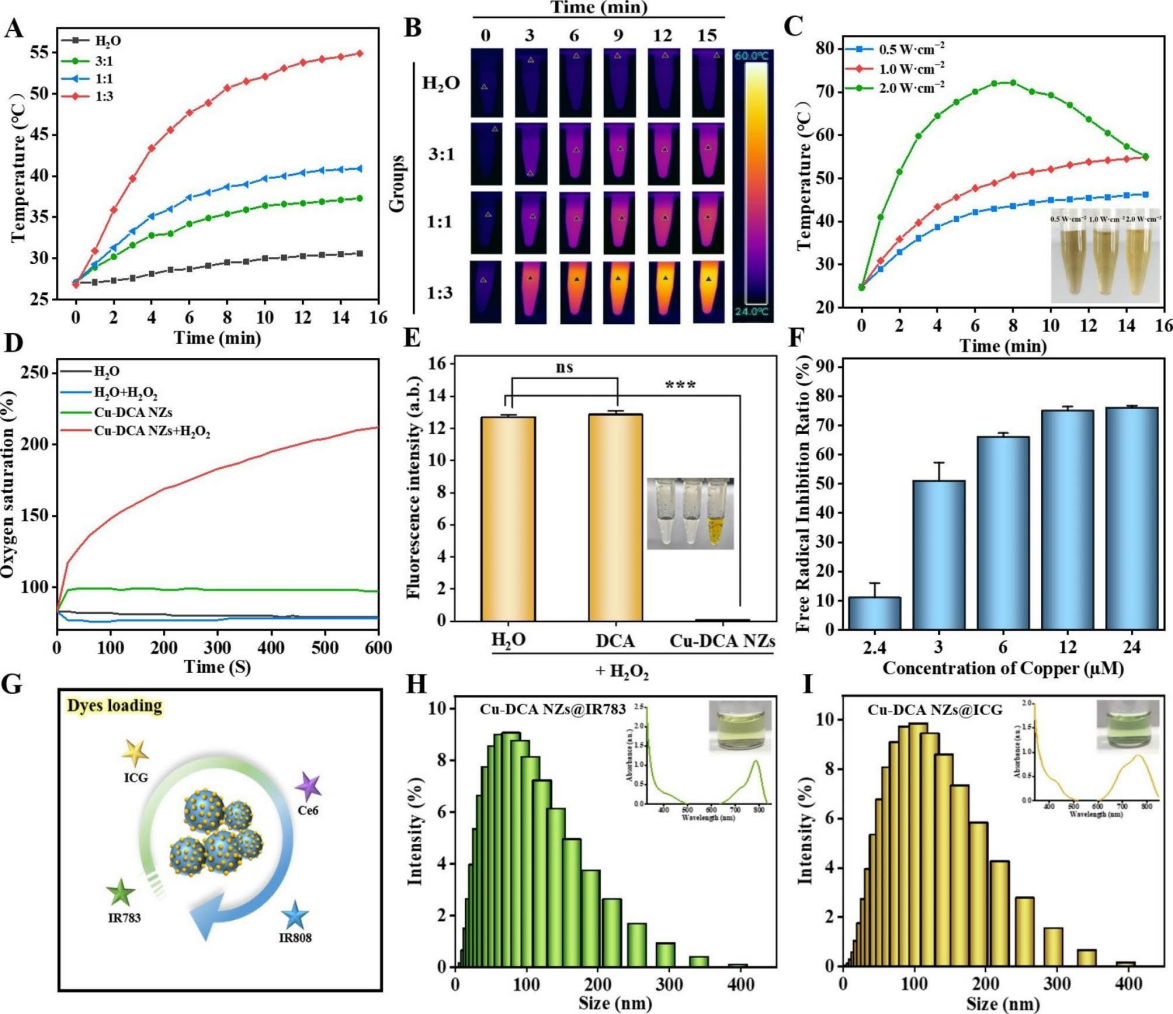
Performances of Cu-DCA NZs. (**A**) Proportion-dependent temperature change curves of Cu-DCA NZs under NIR irradiation (1.0 W cm^− 2^). (**B**) Infrared thermal imaging data of different proportion of Cu-DCA NZs with NIR irradiation (1.0 W cm^− 2^). (**C**) Temperature change of Cu-DCA NZs with NIR irradiation (0.5, 1.0, and 2.0 W cm^− 2^). Inset are different NIR irradiation power solution photographs of Cu-DCA NZs. (**D**) oxygen generation, (**E**) RDPP fluorescence intensity and (**F**) Superoxide anion scavenging ability of Cu-DCA NZs. (**G**-**J**) Drugs loading of Cu-DCA NZs. (**G**) Schematic of drugs loading of Cu-DCA NZs. (**H**-**J**) Size distribution after different drugs loading. Inset are the UV–vis absorption spectra and solution photographs of different drugs. Data are presented as the mean ± SD (n = 3). * *p* < 0.05, ** *p* < 0.01, *** *p* < 0.001 and ns > 0.05

As shown in Fig. 2A, Cu-DCA NZs solutions with reaction molar ratios of 1: 3, 1: 1 and 3: 1 (DCA: Cu(NO_3_)_2_·3H_2_O) displayed proportionally different temperature changes upon NIR irradiation (1.0 W cm^− 2^) at room temperature. The molar ratio of 1:3 was the most significant, with the temperature rising rapidly from 26.8 to 54.9 °C with 15 min of NIR irradiation, while the temperature of the deionized water rose only slightly to 30.4 °C. Therefore, the molar ratio of 1: 3 was chosen for the subsequent studies. At the same time, the images collected from the infrared thermal imager show the temperature changes of the groups intuitively (Fig. 2B). In addition, we compared the temperature changes at different irradiation powers (0.5, 1.0, 2.0 W cm^− 2^), higher temperature at the same point in time as the power increased (Fig. 2C). However, at irradiation powers of 2.0 W cm^− 2^ with 15 min, the temperature of the Cu-DCA NZs solution began to drop after warming up to 72.2 °C and the color of the solution became lighter, suggesting that the thermal tolerance of the Cu-DCA NZs may be lower than 70.0 °C. Moreover, we evaluated both the photothermal stability and photothermal conversion efficiency (η) of Cu-DCA NZs by multiple NIR irradiation, Cu-DCA NZs were irradiated with three cycles (1.0 W cm^− 2^, 15 min per cycle). The results shown that the Cu-DCA NZs has good photothermal stability (Fig. S4) and the photothermal conversion efficiency was determined to be 34.49% according to the corresponding calculation equation (Fig. S5). Besides, considering the side-effect of high temperature on cells (50.0 °C) [39, 40], the final condition chosen was power of 1.0 W cm^− 2^ and irradiation for 5 min (45.6 °C). The results reflected that Cu-DCA NZs has a good photothermal effect and could be used for subsequent related applications.

### Multienzyme-like activities of Cu-DCA NZs

Since the strong reducing properties of sulfhydryl groups and could interact with harmful reactive oxygen species (ROS) to achieve a scavenging effect. At the same time, the change in valence of the copper may also confer some biological activity to Cu-DCA NZs. Therefore, we speculate that Cu-DCA NZs possess some enzymatic activity. There is a balance of oxidation and anti-oxidation in living organisms. Among the biologically relevant ROS, H_2_O_2_ is the most important and common. Therefore, we first investigated the CAT-like activity of Cu-DCA NZs that catalyze the decomposition of H_2_O_2_ (Fig. 2D). A portable oxygen detector was used to measure the oxygen saturation produced by deionized water, DCA (Fig. S6) and Cu-DCA NZs before and after the interaction with H_2_O_2_. From the results shown that Cu-DCA NZs were significantly increasing in oxygen saturation after the addition of H_2_O_2_ compared to the other groups, which indicated that the oxygen content also increased. Whereas there was no significant change in the deionized water group before and after the addition of H_2_O_2_, the DCA group showed a sharp drop after the addition of H_2_O_2_, speculating it was possible that the sulfhydryl groups reacted with the H_2_O_2_. Moreover, the activity of CAT-like activity was measured for different molar ratios of Cu-DCA NZs (Fig. S7) and found that the 1:3 group had the best catalytic activity, further demonstrating the effect of the previously mentioned reaction molar ratio on Cu-DCA NZs. Meanwhile, a comparison of classical Cu-MOF with Cu-DCA (Fig. S8) showed no significant change in oxygen saturation at the same H_2_O_2_ concentration, indicating that Cu-DCA had superior CAT-like activity than classical Cu-MOF. In addition, the generation of oxygen by Cu-DCA NZs with H_2_O_2_ was further verified by the RDPP probe (Fig. 2E). RDPP probes are susceptible to quenching by oxygen, resulting in reduced fluorescence intensity. The results indicating that DCA did not decompose H_2_O_2_, therefore its fluorescence value was not different from that of H_2_O. In contrast, Cu-DCA NZs were able to decompose H_2_O_2_ and production of oxygen to quench the RDPP probe, reducing the fluorescence value.

Furthermore, we further evaluated the SOD-like activity of Cu-DCA NZs using the NBT method [41]. As shown in Fig. 2F, Cu-DCA NZs not only has a good inhibitory effect on superoxide anion (•O^2−^), but also has a concentration dependence. When the concentration is 24 µM, the inhibitory rate is as high as 80.0%. Therefore, Cu-DCA NZs combined simulated SOD and CAT activity in scavenging ROS to achieved the effect of scavenging ROS better.

### Dyes loading properties of Cu-DCA NZs

Based on physisorption experiments confirming the drug loading potential of Cu-DCA NZs, several common fluorescent dyes were selected to co-assembled with Cu-DCA NZs as a means of verifying the drug loading properties of Cu-DCA NZs.

As shown in the scheme Fig. 2G, we loaded with seven different drugs in the Cu-DCA NZs system in sequence, namely four fluorescent dyes IR783, ICG, Ce6 and IR808. As shown in Fig. 2H, I and Fig. S9, the average hydrodynamic sizes of Cu-DCA NZs were increase in particle size from around 20.0 nm to 52.2–72.8 nm from the unloaded after the addition of dyes. Besides, Cu-DCA NZs@IR783/ICG/Ce6/IR808 NPs were found to have the characteristic absorption peaks of each fluorescent dye by UV–vis absorption spectra. And the polymer dispersity index of Cu-DCA NZs was good after loading of different dyes (Fig. S10). The above results reflect the potential of Cu-DCA NZs as MOF-like to be drug-loaded, providing a powerful tool for research and development of treatments for other potential diseases.

### Cytotoxicity and cellular uptake of Cu-DCA NZs in vitro

**Fig. 3:**
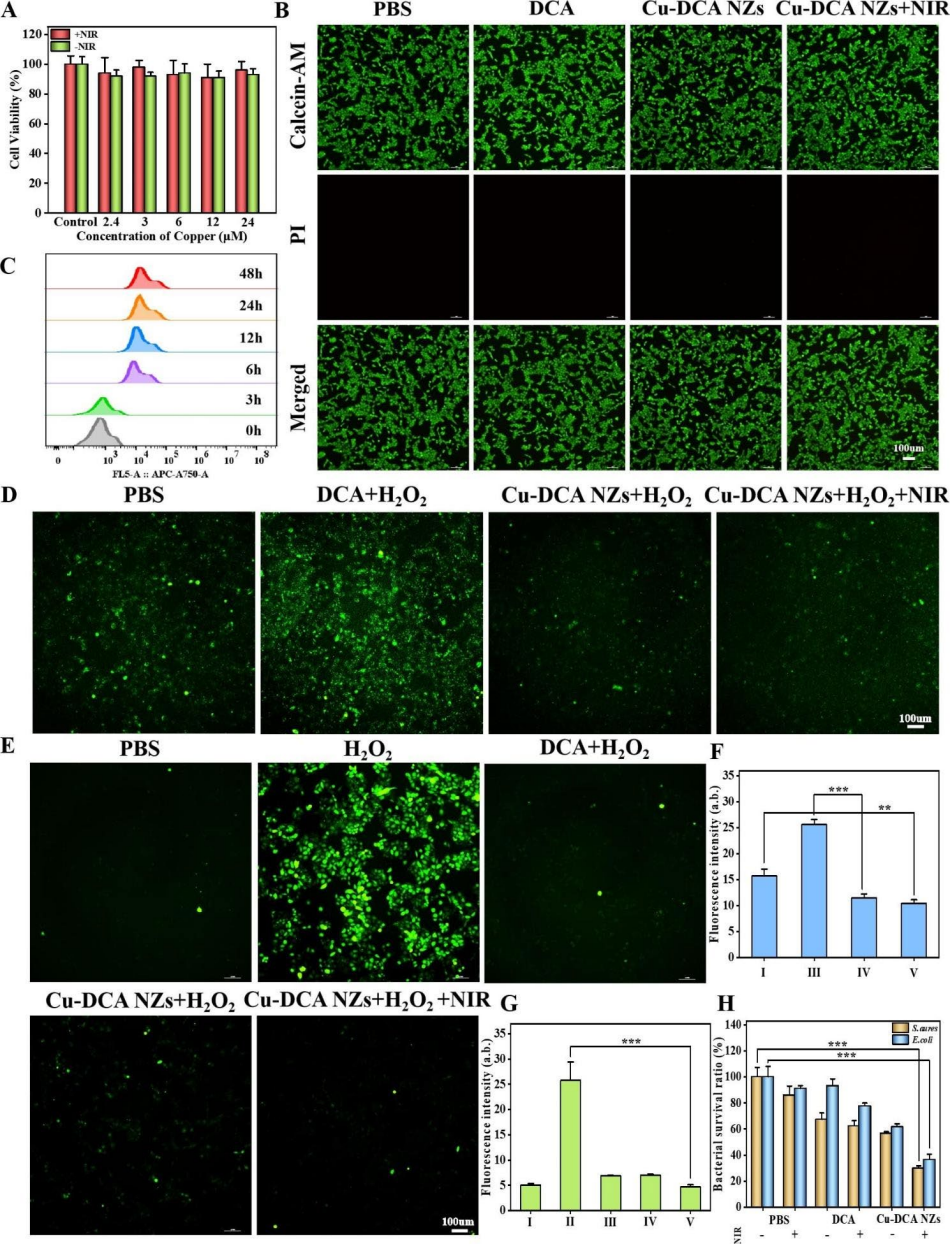
In vitro biocompatibility and antibacterial activity of Cu-DCA NZs. (**A**) The viability of HUVECs after different treatments (1.0 W cm^− 2^, 5 min). (**B**) Calcein/PI cell viability fluorescence images of HUVECs with different treatments (1.0 W cm^− 2^, 5 min). (**C**) Cells uptake of Cu-DCA NZs with different times. (**D**) RDPP probe detecting intracellular oxygen generation level of HUVECs after different treatments. (**E**) DCFH-DA assay detecting intracellular ROS level of HUVECs after different treatments. (**F**) Fluorescence intensity of intracellular oxygen generation level. (**G**) Fluorescence intensity of intracellular ROS level. (I: PBS, II: H_2_O_2_, III: DCA + H_2_O_2_, IV: Cu-DCA NZs + H_2_O_2_, V: Cu-DCA NZs + H_2_O_2_ + NIR). (**H**) The viability of bacterial with different treatments (1.0 W cm^− 2^, 5 min). Data are presented as the mean ± SD (n = 3). * *p* < 0.05, ** *p* < 0.01, and *** *p* < 0.001

At present, research on nanomaterials has reached a consensus to ensure excellent biocompatibility. First, the toxicity of different concentrations of Cu-DCA NZs with or without NIR irradiation for 24 h incubation with HUVECs was assessed. The results of CCK-8 (Fig. 3A) showed that Cu-DCA NZs at different concentrations with or without NIR irradiation did not exhibit significant cytotoxicity and cell survival rates were maintained above 90.0%. Then, Cu-DCA NZs at a concentration of 24 µΜ were finally selected for the subsequent studies. In addition, fluorescence images of Calcein/PI cell viability (Fig. 3B) also confirmed that HUVECs maintained good cell viability after co-incubation with Cu-DCA NZs (24 µΜ), consistent with the results of previous experiments. The results show that Cu-DCA NZs have good biocompatibility.

The level of nanomaterials in cells was noteworthy. For this purpose, the cells uptake of Cu-DCA NZs in HUVECs were investigated by flow cytometry at different time points (0, 3, 6, 12, 24 and 48 h). The HUVECs were treated with Cu-DCA NZs@IR783, and since IR783 was both a photosensitizer and fluorescent substance, Cu-DCA NZs@IR783 required no additional labeling. The results showed that (Fig. 3C and S11) the intracellular fluorescence intensity increased with the extension incubation, which reached highest at 24 h and then went down. Therefore, the optimal incubation time of Cu-DCA NZs was determined to be 24 h, so as to obtain the optimal results in the subsequent experiments.

### ROS scavenging effect of Cu-DCA NZs in vitro

Given the practicalities of using, we chose HUVECs to evaluate their in vitro ROS scavenging effect and to lay the groundwork for subsequent work. Related experiments based on the chemical part demonstrated that Cu-DCA NZs have a strong ROS scavenging effect. To demonstrate that the corresponding activity was maintained on the cells, we first explored whether Cu-DCA NZs are able to regulate intracellular oxygen levels in the presence of cellular hypoxia. After HUVECs were incubated in a hypoxic (1% O_2_) environment and subjected to different treatments, the intracellular oxygen status was verified using RDPP as an oxygen-sensitive probe, whose fluorescence was specifically burst by oxygen molecules, making the fluorescence intensity inversely proportional to the oxygen content. The results of the fluorescence images (Fig. 3D and F) showed a significant decrease in fluorescence intensity in Cu-DCA NZs + H_2_O_2_ group compared to PBS and DCA + H_2_O_2_ groups, indicating that Cu-DCA NZs regulate intracellular oxygen levels and improve cellular hypoxia in the presence of cellular hypoxia. This is further proving that Cu-DCA NZs still have good CAT-like activity in the cells.

Meanwhile, we also chose the DCFH-DA method to determine the changes in ROS levels in each group under co-incubation with H_2_O_2_ (Fig. 3E and G). The addition of H_2_O_2_ stimulates ROS production resulting in a significant increase in fluorescence intensity, whereas the Cu-DCA NZs + H_2_O_2_, Cu-DCA NZs + H_2_O_2_ + NIR and DCA + H_2_O_2_ groups showed a decrease in fluorescence due to SOD-like activity, which resulted in the removal of the ROS produced. It is speculated that it may be the easy oxidation of the sulfhydryl group that confers this excellent ability to scavenge ROS, which is consistent with the phenomenon in chemical experiments.

### Antibacterial effect of Cu-DCA NZs in vitro

Furthermore, there are many reports in the literature on the inhibition of bacterial growth by photothermal therapy, and considering the effect of bacteria on the healing of diabetic wound, Staphylococcus aureus (*S. aureus*) and Escherichia coli (*E. coli*) as the most common bacteria in wound infections were chosen for antibacterial experiments [42]. The antibacterial effect was initially verified by measuring the absorbance at 600 nm of bacterial suspensions. As shown in Fig. 3H, compared to the PBS, the Cu-DCA NZs had some antibacterial effect, probably stemming from the copper ions present. However, the bacterial survival rate was still as high as 50.0% before NIR irradiation. After NIR irradiation (1.0 W cm^− 2^, 5 min), the bacterial survival rate of *S. aureus* and *E. coli* could be reduced to 30.1% and 36.6% respectively. This suggests that the photothermal effect produced by Cu-DCA NZs after NIR irradiation induces bacterial death and thus inhibiting bacterial growth, but the principle of inhibition remains to be explored. The antibacterial effect shown by Cu-DCA NZs in vitro has potential for subsequent application in vivo.

**Fig. 4:**
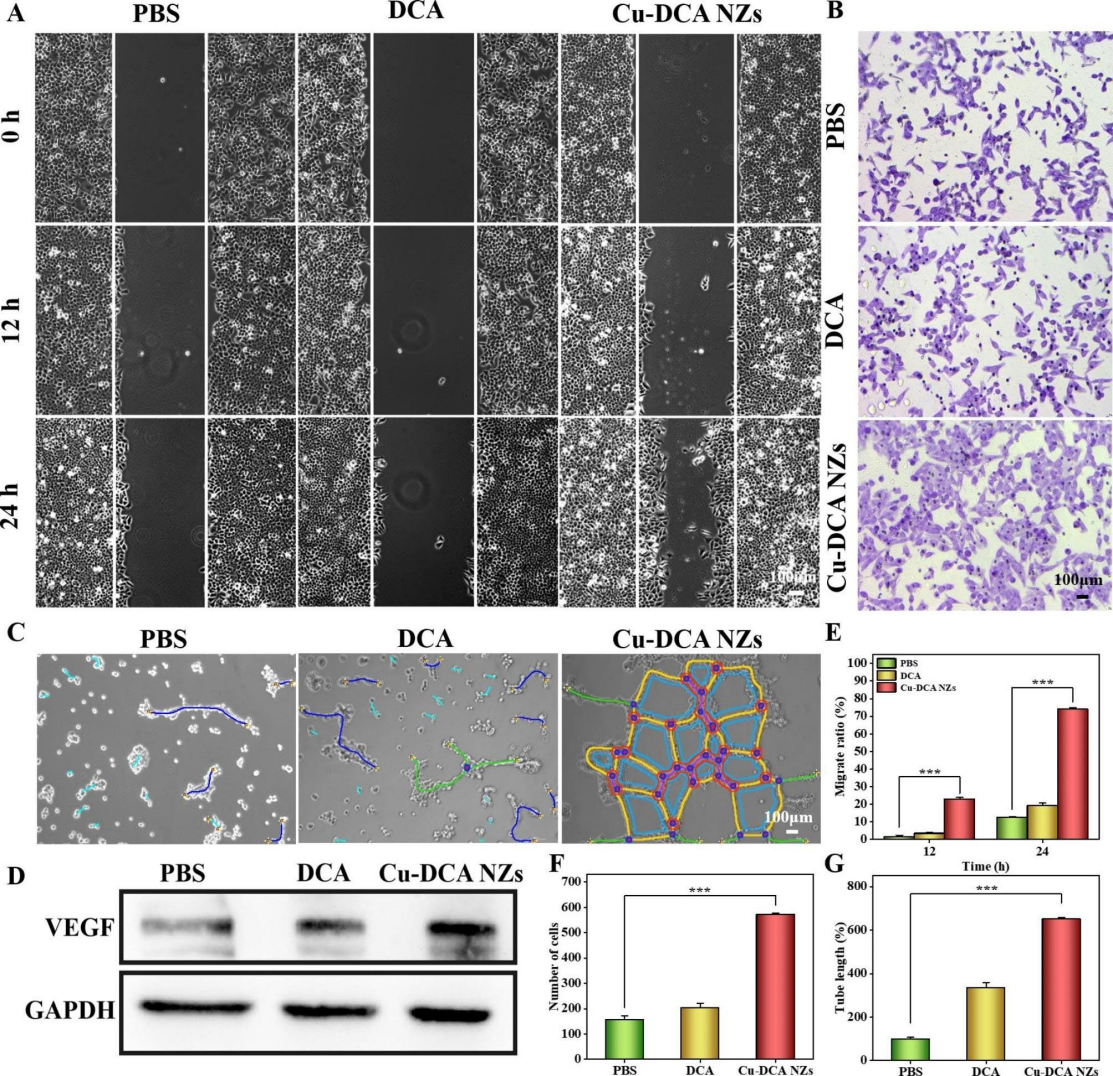
In vitro angiogenesis study of Cu-DCA NZs. (**A**) Images of HUVECs migration with different treatments for 0, 12, and 24 h. (**B**) Images of HUVECs migration with different treatments in transwell migration assay. (**C**) Images of tube formation with different treatments. (**D**) Western blot analysis of VEGF expression levels. GAPDH served as an internal reference. (**E**) The HUVECs migration ratio with different treatments. (**F**) Number of cells passing through the membrane in the transwell migration assay. (**G**) Total length of the tube network in tube formation assay. Data are presented as the mean ± SD (n = 3). * *p* < 0.05, ** *p* < 0.01, and *** *p* < 0.001

### Evaluation of angiogenesis effects of Cu-DCA NZs in vitro

Angiogenesis is a key factor in wound healing. It has been reported that copper ions can promote angiogenesis by inducing the production of VEGF, thereby promoting wound healing. Thus, we validated the ability of Cu-DCA NZs to induce angiogenesis in vitro. Considering that copper ions are a major factor in promoting cell migration and angiogenesis, three experimental groups were set up for follow-up experiments: PBS, DCA, Cu-DCA NZs. First, scratch experiments (Fig. 4A and E) showed the cell migration of HUVECs after different treatments. As shown, after 12 and 24 h of incubation, the Cu-DCA NZs group significantly promoted cell migration compared to the PBS and DCA groups, which also indicated that the release of copper ions was the main reason for promoting cell migration. Then, the transwell migration assay showed (Fig. 4B and F) that there was a clear tendency for the cells to across the membrane after the treatment of Cu-DCA NZs, which is consistent with the results of the scratch assay.

Afterwards, the in vitro tube formation ability of Cu-DCA NZs was then further evaluated (Fig. 4C, G and S12). As shown, the Cu-DCA NZs group significantly promoted a marked increase in tubule length and increased tubule connectivity compared to the PBS and DCA groups. In contrast, the PBS and DCA groups did not show much more differences in tubule network formation. To further reveal the role of Cu-DCA NZs on angiogenesis, we assessed the expression of VEGF in HUVECs after different treatments. As shown in Fig. 3D and S13, the expression levels of VEGF in the Cu-DCA NZs group were notably higher than the PBS and DCA groups. Taken together, the above results suggest that promoting the migration of HUVECs as well as angiogenesis in vitro by upregulating the expression of VEGF by Cu-DCA NZs, which is beneficial to the angiogenesis and recovery of diabetic wound.

### Diabetic wound therapeutic effect of Cu-DCA NZs in vivo

**Fig. 5:**
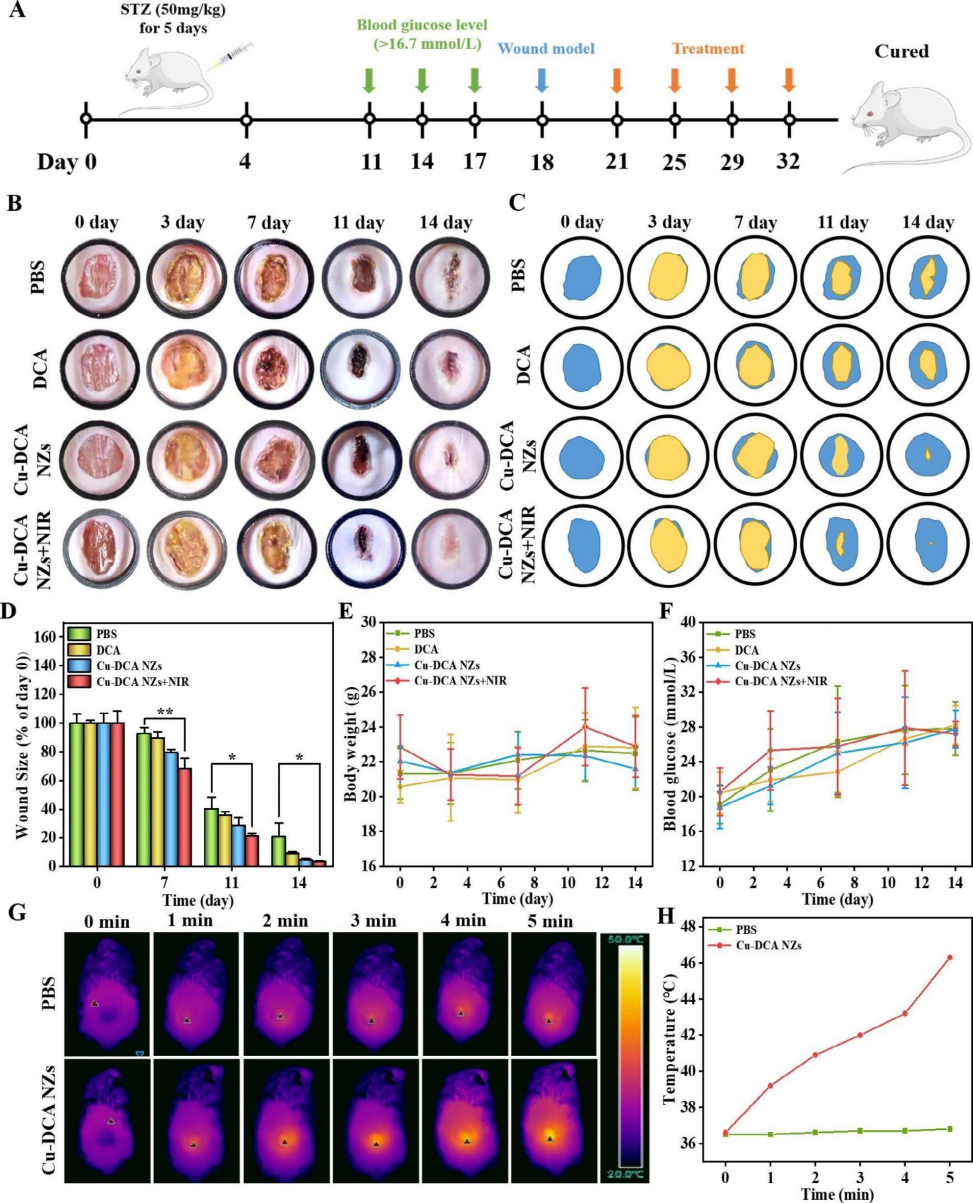
In vivo diabetic wound treatment studies. (**A**) Schematic of diabetic wound treatment. (**B**) Representative photographs of the wound treated with different treatments for 14 days. (**C**) Simulated images of wound healing in different treatment groups. (**D**) Wound size change during 14 days. Wound size at each time point was normalized to day 0. (**E**) Body weight of diabetic mice in different groups during treatments. (**F**) Blood glucose of diabetic mice in different groups during treatments. (**G**, **H**) Representative real-time thermographic images with NIR irradiation (1.0 W cm^− 2^, 5 min) and the corresponding photothermal heating curves. Data are presented as the mean ± SD (n = 3). * *p* < 0.05, ** *p* < 0.01, and *** *p* < 0.001

Encouraged by the in vitro effects of Cu-DCA NZs on ROS scavenging and cell migration, we tested the healing effect of in vivo treatment by treating total excisional wound in diabetic mice. The treatment strategy was shown in Fig. 5A, diabetes was induced by intraperitoneal injection of streptozotocin (STZ) into Balb/c mice, and the blood glucose levels were monitored for a fortnight until they exceeded 16.7 mmol/L to confirm successful molding. Then a diabetic mouse model of total dorsal excision wound was further established and the mice were randomly divided into 4 groups (PBS, DCA, Cu-DCA NZs and Cu-DCA NZs + NIR) to observe the effect of different treatments on the healing of diabetic wound. At the end of the treatment, the wound area was quantified using ImageJ software and the wound healing in each group at different times is shown in Fig. 5B and C. During the first 3 days of treatment, there was no significant change in the size of the wound in each group for the time being. By day 7 of treatment, the Cu-DCA NZs + NIR group had a more intact scab and a significant reduction of 31.9% in the area of the healed wound compared to the PBS and DCA groups where infection and bleeding were still present (Fig. 5D). And the Cu-DCA NZs + NIR group continued to have a good therapeutic effect in the follow-up treatment. The quantitative results further showed that at the end of 14 days of treatment, the wound in the Cu-DCA NZs + NIR group were largely healed, with a reduction in wound area to 3.40%, while the control group wound remained unhealed, with a wound area of 20.8%. The above results indicate that the Cu-DCA NZs + NIR group could significantly promote the healing of diabetic wound. In addition, the body weight and blood glucose levels of the mice in each group were continuously monitored during the treatment period, and there was no significant change in body weight (Fig. 5E), and the blood glucose level remained above 16.7 mmol/L throughout the treatment period, indicating that the mice were always in a diabetic state (Fig. 5F).

Simultaneously we evaluated the temperature trends of Cu-DCA NZs in vivo under NIR irradiation. As shown in Fig. 5G, the thermal imaging camera monitored the temperature changes of PBS and Cu-DCA NZs under NIR irradiation (1.0 W cm^− 2^, 5 min). the temperature of Cu-DCA NZs group gradually increased from 36.6 to 46.3 °C after 5 min of irradiation, while the temperature of the PBS group only changed slightly from 36.5 to 36.8 °C (Fig. 5H). This indicated that Cu-DCA NZs also had a significant photothermal effect in vivo. In addition, considering the highly efficient antibacterial effect of Cu-DCA NZs in vitro under NIR irradiation, we supplemented a mouse model of diabetic infected wound to assess the antibacterial ability in vivo. And antibacterial effect was further observed by photographic recording as well as agar plates coating. The results (Fig. S14) showed that the Cu-DCA NZs + NIR group had the highest antibacterial effect compared to the other groups, which was consistent with the in vitro results and also provided a protection for wound healing. The above results suggested that Cu-DCA NZs exert photothermal effects under NIR irradiation to inhibit bacterial growth on wound for accelerating the wound healing.

### Histological and immunohistochemical analysis

**Fig. 6:**
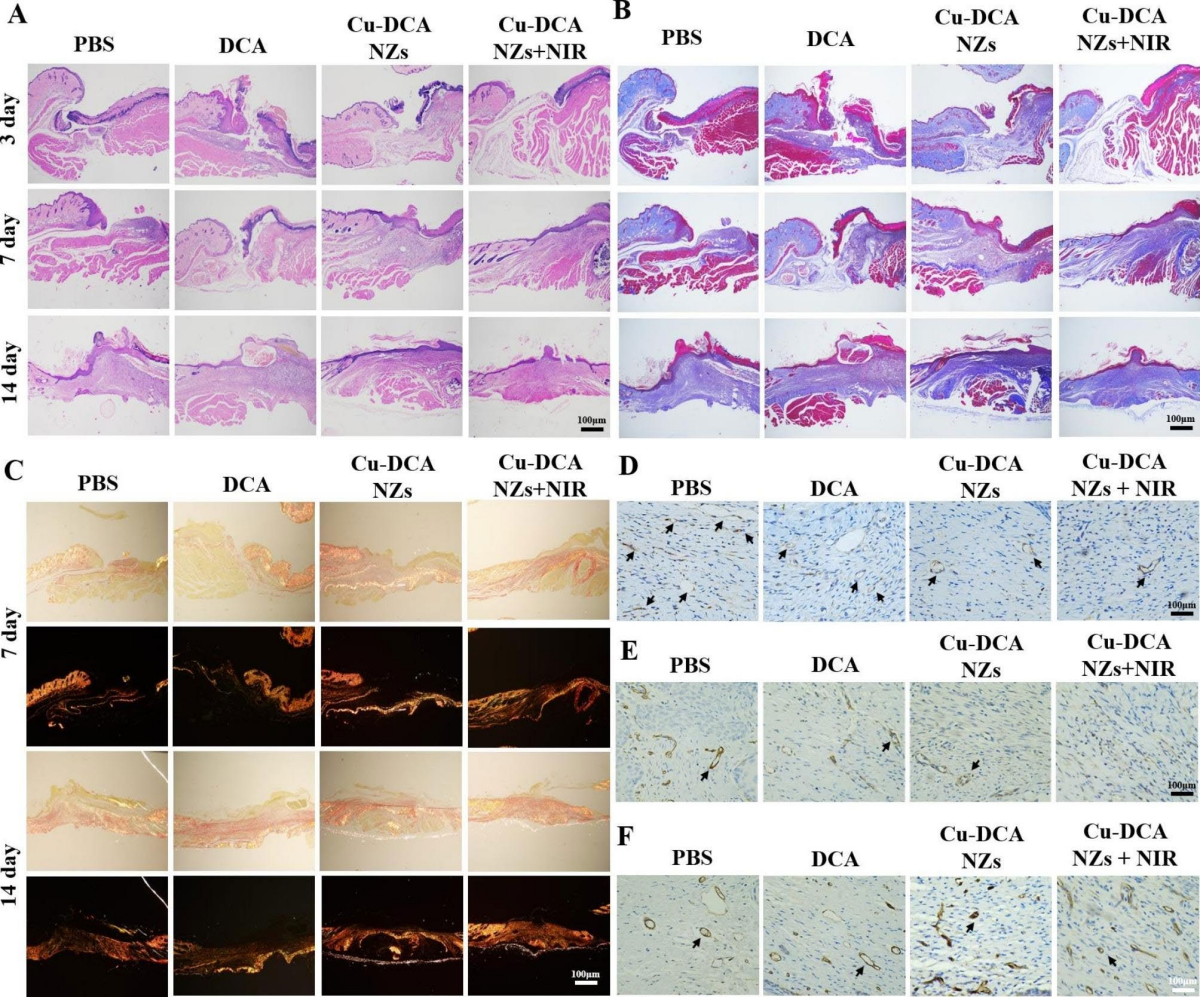
Histological analysis in wound healing. (**A**, **B**) Representative H&E staining and Masson’s trichrome staining images of skin tissues after different treatments. (**C**) Representative Picrosirius Red and cross-polarized optical microscope images of wounded tissue after different treatments at days 7 and 14. (**D**-**F**) Representative images of VEGF, α-SAM and CD31after different treatments at days 14. (The black arrow indicates the region of positive expression.)

To further assess the wound healing in diabetic mice, H&E staining was performed on each group (Fig. 6A). After 3 days of treatment, granulation tissue started to form in the Cu-DCA NZs + NIR group, while the wound border between the wound and normal tissue was clear and well defined in the other treatment groups. As shown in Fig. 6A, new epithelial structures were evident in the wound of mice treated with Cu-DCA NZs + NIR at 7 days after treatment, and the size of the wound was reduced. In contrast, the PBS and DCA groups still had some degree of infection and abscesses at the end of 14 days of treatment, with delayed healing progress. Collagen fiber deposition was also assessed by Masson’s trichrome staining of the tissue. As shown in Fig. 6B, the filamentous collagen fibers were stained blue-purple. In the early stage of wound healing, the wound collagen fiber in the Cu-DCA NZs + NIR group started to form and gradually matured and precipitated in a more tightly ordered arrangement 14 days after treatment, which was consistent with the trend of H&E staining. In addition, we assessed the deposition of collagen fibers on the wound by Sirius red staining (Fig. 6C). Under a cross-polarized optical microscope, the orange-red, tightly arranged were mature collagen type I fibers. While the blue-green, filamentous and sparsely distributing were new collagen type III fibers [43]. As shown in Fig. 6C, on day 7 of treatment, mature type I collagen fibers began to establish on the wound and gradually replaced the new type III collagen fibers in the Cu-DCA NZs + NIR group, suggesting an accelerated repair process. After 14 days of treatment, it was observed that the mature type I collagen fibers in the Cu-DCA NZs + NIR group were more widely distributed and more closely aligned compared to the other treatment groups, indicating a better healing effect. The above results shown that the Cu-DCA NZs + NIR group could promote the growth of wound epidermis and the deposition of collagen to promote healing.

As angiogenesis is critical to the repair of diabetic wound, to further explore its effect on healing, VEGF expression levels were evaluated by immunohistochemical staining for assessing the density of vessels (Fig. 6D and S15). VEGF expression is low in normal skin tissue and rises in response to tissue damage, gradually returning to normal levels as healing progresses. On day 14, VEGF expression was significantly lower in the Cu-DCA NZs + NIR group than other treatment groups [44], which may be due to the completion of the growth of new blood vessels in the wound treated in the Cu-DCA NZs + NIR group. In contrast, the other treatment groups had higher VEGF expression levels as the vessels were still in the process of regeneration, suggesting that the wound in the Cu-DCA NZs + NIR group have gradually returned to normal in the later stages of wound repair. Meanwhile, immunohistochemical staining for the vascular smooth muscle marker alpha-smooth muscle actin (α-SMA) showed the lower expression in the Cu-DCA NZs + NIR group than other treatment groups on day 14 (Fig. 6E and S16), indicating that no proliferating scar tissue in the wound and the healing progress was well [45]. Then, as platelet endothelial cell adhesion molecule-1 (CD31) could probe the degree of vascularisation of wound tissue, its positive expression rate is one of the most vital markers of wound vascular microcirculation. The expression levels of the angiogenic marker CD31 were continued to be evaluated by immunohistochemical staining, further validating the pro-angiogenic effect of Cu-DCA NZs + NIR in vivo. The immunohistochemical staining images of CD31 in Fig. 6F and S17 showed that the Cu-DCA NZs + NIR group had more neovascularisation while the other treatment groups had less neovascularisation. And after quantifying the positive expression of CD31 in each group, it was found that CD31 expression was significantly increased in the Cu-DCA NZs + NIR group compared to the PBS group. The above results suggested that Cu-DCA NZs + NIR could accelerate wound healing by promoting angiogenesis and reducing scar proliferation.

Moreover, based on Cu-DCA NZs with CAT and SOD-like activities and in photothermal effects of antibacterial, we assessed the inflammatory profile of the wound by immunohistochemical staining for a number of common pro-inflammatory cytokines, including interleukin 6 (IL-6), interleukin 1β (IL-1β) and tumor necrosis factor-α (TNF-α). As shown in the Fig. S18, the Cu-DCA NZs + NIR group had the lowest inflammation expression, indicating its significant anti-inflammatory effect. The above results indicate that the Cu-DCA NZs + NIR group can effectively reduce the accumulation of wound inflammation and have a positive effect on wound repair. Taken together, these histological and immunostaining results suggest that Cu-DCA NZs can effectively promote diabetic wound healing by promoting wound angiogenesis and collagen fibers deposition under NIR irradiation, while scavenging ROS to reduce inflammation levels.

To assess the in vivo toxicology of Cu-DCA NZs + NIR, H&E staining was performed on the major organs of mice, including heart, liver, kidney, lung and spleen (Fig. S19). After comparison with the PBS group, no damage or pathological changes were observed in the major organs in the Cu-DCA NZs + NIR group. In addition, the results of blood routine examination and biochemical indicators showed that the values of each indicator were within the normal range (Fig. S20 and S21). All the above results indicated that Cu-DCA NZs have good biosafety.

## Discussion

Chronic poor skin wound healing due to persistent hyperglycaemia is a serious complication of diabetes [46]. Compared to the healing process of normal wounds, diabetic wounds are marked by impaired healing, limited oxygen supply to the wound, neovascular obstruction, slow and persistent inflammation, and acute bacterial infection [47]. However, due to the complex pathological microenvironment of diabetic wounds, the lack of synergistic treatment or versatile strategies in clinical practice leads to increased mortality and disability rates [48]. Therefore, there is an urgent need to develop new multifunctional treatment strategies to promote the healing of diabetic wounds.

In previous studies, copper ions have been shown to have great potential in promoting cellular angiogenesis. However, the concentrated release of copper ions may lead to excessive local concentrations and cause toxicity. In recent years, several studies have shown that metal-based organic complexes may exhibit better biological properties [49] and Cu-MOF nanoparticles have been widely used in studies of chronic wound healing. But, the large and unstable size of Cu-MOF particles and their easily damaged structure maked it difficult to ensure controllability during treatment and lack the ability to regulate the complex microenvironment of diabetic wounds. Meanwhile, the emergence of nanozymes has become a research hotspot in recent years. Due to their high structural stability, adjustable catalytic activity and diverse functions, nanozymes confer great demand for applications in biomedicine and industrial catalysis [50]. For example, inspired by glucose metabolism, Shan et al. [51] proposed a microneedle patch with dual nanozyme activity to achieve in situ oxygen generation and alleviate traumatic hypoxia by mimicking CAT activity. Abhishek Sahu et al. [52] found that prussian blue nanozymes not only degrade hydrogen peroxide but also have a powerful superoxide scavenging capacity as a means of reducing excess ROS and regulating inflammation for wound healing. Benefit from the development of nanozymes, there is a greater variety of materials used for wound repair, while there are few reports of diabetic wounds healing based on the copper nanozymes. Therefore, it may be more beneficial for the synergistic repair treatment of diabetic wounds if more functions may be given to copper nanozymes while maintaining their intrinsic enzymatic activity.

Based on the above considerations, inspired by the co-assembly strategy, we have prepared a multifunctional nanozyme for the synergistic treatment of diabetic wounds. In this study, we prepared uniformly size-controllable nanozymes Cu-DCA NZs by simple co-assembly of the antioxidant organic dual ligand molecules DCA and copper ions. Under NIR irradiation, Cu-DCA NZs could effectively inhibit the growth of wounds bacteria and reduce the extent of infection through photothermal therapy. At the same time, due to Cu^2+^/Cu^+^ doping on the surface of Cu-DCA NZs, it exerts an effective mimicry of SOD and CAT activities, which not only catalyzed the conversion of intracellular H_2_O_2_ to oxygen thereby alleviating wound hypoxia, but also improved inflammation accumulation. Furthermore, the sustained release of copper ions accelerates cell proliferation, migration and angiogenesis, further effectively promoting diabetic wound healing. More importantly, in addition to the above multiple properties, Cu-DCA NZs also have the potential to be loaded with other drugs and may play a greater role in the future in areas related to skin regeneration and other oxidative stress diseases.

## Conclusion

In summary, we have developed a multifunctional Cu-DCA NZs for diabetic wound healing. On the basis of multitopic carboxylic acids used to construct metal-organic frameworks, a dual ligand molecule DCA containing both carboxyl and sulfhydryl groups were co-assembled with copper ions to take multifunctional Cu-DCA NZs with uniform size and multiple enzyme-mimetic activities. Meanwhile, Cu-DCA NZs have potential for drug delivery under simple co-assembly the dye molecules. In addition, in the process of wound healing, Cu-DCA NZs can inhibit bacterial growth under NIR irradiation, reduce inflammation and regulate oxygen balance by promoting blood vessel growth, collagen fiber deposition, and jointly significantly promote wound healing of diabetes. In vitro and in vivo experiments have shown that Cu-DCA NZs display good safety profiles. Thus, the developed Cu-DCA NZs has considerable potential for the future development of chronic wound repair.

## Methods

### Materials

Copper(II) nitrate hydrate (Cu(NO_3_)_2_·3H_2_O), 2,5-dimercaptoterephthalic acid (DCA), IR 783, IR 808, Chlorin e6 (Ce6), Indocyanine green (ICG), Tris(2,2-bipyridine) ruthenium chloride (RDPP), H_2_O_2_ (technical grade, 30%) were provided by Sigma–Aldrich (USA). Nitrotetrazolium blue chloride (NBT) were purchased from TCI (Japan). The CCK-8 kit was purchasedfrom MCE (USA). A Tris-HCl (pH = 8.8, 1 M), anhydrous (NaCO_3_), BCA protein concentration determination kit, Calcein/PI cell viability/cytotoxicity assay kit, reactive oxygen detection kit, crystal violet were obtained from Beyotime (China). Matrigel® Matrix was purchased from Corning (USA). Penicillin–streptomycin solution (100X), foetal bovine serum (FBS), trypsin, Dulbecco’s modified Eagle’s447 medium (DMEM) were obtained from Gibco (USA). Phosphate-buffered saline (PBS, pH = 7.4) solution was prepared in the laboratory. All chemicals and reagents were of analytical grade and used as received without further purification. Millipore deionized water (18.2 MΩ · cm) was used for all experiments.

### Synthesis of Cu-DCA NZs

The Cu-DCA NZs were prepared by a one-pot synthesis method. Briefly, Cu(NO_3_)_2_·3H_2_O (15 mg) and DCA (5 mg) were dissolved in 1 mL 0.2 M Tris-HCl separately. Then, Cu(NO_3_)_2_·3H_2_O solution was added slowly to the above DCA solution. The reaction mixture was at room temperature for 4 h with constantly stirring. After the reaction, the product was purified by dialysis with deionized water for 24 h.

### Characterization of Cu-DCA NZs

The micromorphology of Cu-DCA NZs was observed through Atomic force microscope (AFM) (SPM-9700, SHIMADZU, Japan) and Transmission electron microscope (TEM) (Talos F200S, FEI, USA). Dynamic light scattering (DLS) was used to characterize the particle size, polydispersity index (PDI) and surface zeta potential of the relevant samples by Malvern Instruments (ZEN3600, Zetasizer Nano ZS, UK). Automated Surface Area and Porosity Analyzer (BET) was used to detected nitrogen sorption isotherm and the corresponding pore size distribution (ASAP 2460/ TriStar2 3020/ 3flex, Micromeritics, USA). Ultraviolet-visible (UV–Vis) absorption spectra was detected by UV–Vis spectrophotometer (Nicolet iS5, Thermo Fisher Scientific, USA). Fourier transform infrared (FTIR) spectra was detected by FTIR spectrometer (Nicolet iS5, Thermo Fisher Scientific, USA). X-ray Powder diffractometer (XRD) pattern was detected by XRD (D8 ADVANCE, Bruker, GER). X-ray Auger electron spectroscopy (XPS) measurement were performed by an ESCALAB 250 Xi (Thermo Scientific, USA) X-ray resource. The concentration of Cu was detected by inductively coupled plasma-atomic emission spectrometry (ICP-OES, Agilent 730, USA). The concentration of Cu-DCA NZs used in following studies is calculated based on Cu element.

### Photothermal properties measurement

The photothermal conversion ability of Cu-DCA NZs was investigated by monitoring the temperature rise with a NIR irradiation. The 1.5 mL eppendorf tubes was filled with 1 mL water or Cu-DCA NZs solution with different rates (DCA: Cu(NO_3_)_2_·3H_2_O = 3:1/ 1:1/ 1:3) and exposed to laser for 15 min (808 nm, 1 W·cm^− 2^). Specifically, the photothermal stability was implemented by placing Cu-DCA NZs solution (DCA: Cu(NO_3_)_2_·3H_2_O = 3:1) under irradiation with three repeated cycles of 15 min irradiation ON and 15 min OFF. In addition, an infrared thermal imager (FOTRIC 268D, China) was used to take images of the above solutions under NIR irradiation (808 nm, 1 W·cm^− 2^). The photothermal conversion efficiency (η) was calculated from (1), (2), and (3). ƮS is the time constant of thermal performance. Cd and Md represented the heat capacity and quality of the solution system, respectively. Q_0_ is the background energy. T_water_ and T_surr_ represent the highest rising temperature of the water and the current ambient temperature, respectively. T_max_ is the maximum temperature of Cu-DCA NZs. I and A808 nm are the power of 808 nm laser and the absorbance of Cu-DCA NZs, respectively. At last, the value of photothermal conversion efficiency (η) can be obtained by the (1) Equation.

(1) η = [hS(T_water_-T_surr_)-Q_0_]/I(1-10^A808^).

(2) ƮS = MdCd/hS.

(3) Q_0_ = hS(T_water_-T_surr_).

### Oxygen generating activity study

The ability of Cu-DCA NZs to catalyse H_2_O_2_ mimicking CAT was investigated by monitoring the production of oxygen. A portable oxygen detector (JPBJ-608, INESA, China) was utilized to measure the real-time oxygen level. The deionized water (2ml) or Cu-DCA NZs solution was poured into a 10 ml centrifugal tubes and constantly stirred at room temperature. The deionized water was used in the control group. Then H_2_O_2_ (100 mM, 20 µL) was added and monitors oxygen production at 10 s intervals.

The RDPP probe experiment was according to the reported method [53]. First, 200 µL H2O2 (0.8 M) was mixed well with 200 µL of different groups (H_2_O, DCA and Cu-DCA NZs). Then, 2 µL RDPP probe (2.0 mM) was added to the solution. The mixture was shaken at 37 ℃ for 30 min and finally measured by Varioskan LUX microplate reader. Ex = 455 nm, Em = 613 nm.

### SOD-like activity study

The SOD-like activity of Cu-DCA NZs was determined by Nitrotetrazolium Blue chloride (NBT) method. The detection solution was prepared according to the literature: NBT (2.0 mM), ethylene diamine tetraacetic acid (EDTA, 0.1 M) and riboflavin (1.2 mM) were dissoived in PBS (pH 7.8, 10 mM). Then, 100 µL riboflavin was added into the solution which containing 150 µL NBT, 400 µL EDTA and 5.8 mL PBS, and the mixture was used as detection solution. Cu-DCA NZs solution with different concentrations (50 µL) was mixed with 100 µL of detection solution (n = 6) in 96-well plates. The mixture was shaken at 37 °C for 5 min followed by illumination for 3 min using a 0.2 W light tube. The excessive superoxide free radical which could not be inhibited by Cu-DCA NZs would react with nitroblue tetrazolium to generate a blue product, which could be detected at 560 nm. The inhibition rate was calculated by the decrease of absorbance at 560 nm.

### Dyes loading properties study

The adding procedure is as described above. After slowly adding Cu(NO_3_)_2_·3H_2_O to the system and mixing uniformly, Ce6/ IR 783/ ICG/ IR 808 solution (dissolved in DMSO respectively, 10 mM, 100 uL) was added dropwise to the mixture solution. Then the mixture was constantly stirring at room temperature for 4 h. After the reaction, the purification procedure was the same as for Cu-DCA NZs. The products were collected and dialyzed in deionized water for 24 h for purification, and the particle size was characterized by DLS.

### Cell culture

For cell culture experiments, human umbilical vein endothelial cells (HUVECs, Sciencell, USA) were cultured in DMEM medium supplemented with FBS (10%), penicillin (100 U·mL^− 1^) and streptomycin (100 U·mL^− 1^) in a humidified incubator supplied with 5% CO_2_.

### Cytotoxicity study

The cytotoxicity of Cu-DCA NZs was determined with CCK-8. Classified as dark and phototoxic. The cultured HUVECs were seeded into each well of 96-well plates with density of 5 × 10^3^ cells in 100 µL of DMEM medium 24 h prior to the experiment. Then, 5 µL of solution containing Cu-DCA NZs at a copper concentration varying from 2.4 to 24 µM was added (n = 6) and incubated with cells for another 24 h. (Phototoxic of Cu-DCA NZs was irradiated under a laser (808 nm, 1.0 W·cm^− 2^, 5 min) before 24 h of incubation). The cells added with PBS were used as a control group. At the end of the incubation, 10 µL of CCK-8 was added to each well and the plates were incubated for 1 h at 37 °C. Finally, the absorbance at 450 nm was measured using a Varioskan LUX microplate reader for viability analysis. Meanwhile, the viability of group PBS, DCA, Cu-DCA NZs and Cu-DCA NZs + NIR were evaluated using Calcein/PI cell viability/cytotoxicity assay kit.

### Cell uptake study

To assess the cellular uptake of Cu-DCA NZs, HUVECs were seeded onto 6-well plates with a density of 5 × 10^4^ cells per well. and incubated in a cell incubator for 24 h. Then, Cu-DCANZs@IR783 was added to each well at different times (0, 3, 6, 12, 24, 48 h), and the cells in each well were uniformly washed with PBS. The cells were resuspended in 200µL of PBS after trypsin digestion, and their fluorescence intensity was measured by flow cytometry (Thermo Fisher Scientific, USA).

### In vitro antibacterial experiments

Gram-positive bacteria *Staphylococcus aureus* (*S. aureus*) and Gram-negative bacteria *Escherichia coli* (*E. coli*) were purchased from the Agricultural Re- sources Planning Institute of the Chinese Academy of Agricultural Sciences. The bacteria were cultured overnight in a Luria-Bertani (LB) medium at 37 °C. The antibacterial effect of Cu-DCA NZs was evaluated by absorbance detection. Firstly, 50 µL of samples (PBS, DCA and Cu-DCA NZs solution) was added to 1mL of bacterial suspension (10^8^ CFU·mL^− 1^) and incubated at 37 °C for 24 h. Then, the absorbances of the bacterial suspensions containing the different samples were detected at 600 nm. The relative viability of bacteria is calculated by the following formula:

Relative viability (%) = (Sample - blank) / (PBS - blank) ∗100%. (Blank is the absorbance values of LB medium).

### In vitro ROS scavenging experiments

Briefly, HUVECs were seeded onto 6-well plates with a density of 5 × 10^4^ cells per well. After 24 h incubation, except of the control group and Cu-DCA NZs group, H_2_O_2_ (150 µM) was added to the wells. Subsequently, the samples (PBS, DCA and Cu-DCA NZs solution) were immediately added for incubation of 24 h. Then, the group of Cu-DCA NZs + H_2_O_2_ + NIR wells was exposed to laser irradiation (808 nm, 1 W·cm^− 2^, 5 min). After irradiation, the DCFH-DA probe was added according to the instructions of the reactive oxygen detection kit and incubated for 30 min. Finally, the fluorescence images were taken by a Nikon Eclipse Ti-S inverted fluorescence microscope (Nikon Corporation, Japan).

### In vitro oxygen generating activity experiments

To demonstrate the production of oxygen in cells, the oxygen probe RDPP was used to detect the amount of oxygen in the cells. Firstly, HUVECs were seeded onto 6-well plates with a density of 5 × 10^4^ cells per well and incubated 24 h, followed by the addition of RDPP dye (2 µM) and co-incubation with the cells for 4 h. Then, the samples (PBS, DCA and Cu-DCA NZs solution) were further incubated for 24 h. After 24 h incubation, H_2_O_2_ (150 µM) was added to per wells for incubation of 0.5 h. Finally, the fluorescence images were taken by a Nikon Eclipse Ti-S inverted fluorescence microscope (*E*_*X*_ = 455 nm, *Em* = 613 nm). According to the instructions, RDPP is a fluorescent dye with green fluorescence and quenched by oxygen, and the stronger of fluorescence the lower of oxygen content in the cells.

### Scratch and migration assays

Firstly, HUVECs were seeded onto 6-well plates with a density of 8 × 10^4^ cells per well and incubated with the samples (PBS, DCA and Cu-DCA NZs solution) for 24 h. After 24 h incubation, the cell layer formed in the well plate was scratched with a pipette tip, and the crawling of cells was taken by inverted fluorescence microscope at 0, 12 and 24 h.

For the migration assay, HUVECs were inoculated into the upper chamber of transwell chambers (Corning, USA) and the lower chamber was added to different groups of samples (PBS, DCA and Cu-DCA NZs solution). After 24 h incubation, the chambers were removed and washed. Then fixed with paraformaldehyde for 15 min, then stained with crystal violet for 40 min. Finally the migration of cells were observed under an orthomosaic microscope (Jnnovel, China).

### Tube formation assays

HUVECs were added into 24-well plates with a density of 2 × 10^4^ cells per well and incubated with the samples (PBS, DCA and Cu-DCA NZs solution) for 24 h. Afterwards, Matrigel (Corning, USA) was slowly added onto the 48-well plates of 150 µL per well, and solidified at 37 ℃ for 30 min. The above treated cells were then collected and plated on the 48-well plate and incubated at 37 ℃. Finally, the enclosed mesh of complete microtubes were taken by the inverted fluorescence microscope and quantified by Image J software.

### Western blotting analysis of VEGF

A western blot assay (WB) was selected to evaluate the in vitro angiogenesis effects of Cu-DCA NZs in HUVECs. In brief, HUVECs were seeded onto 6-well plates with a density of 8 × 10^4^ cells per well and incubated with the samples (PBS, DCA and Cu-DCA NZs solution) for 24 h. HUVECs were collected after different treatments, and intracellular proteins were extracted with RIPA lysis buffer on ice. The protein concentration was determined using a bicinchoninic acid protein detection kit (Biosharp, China). The proteins were separated by sodium dodecyl sulfate–polyacrylamide gel electrophoresis and transferred to a polyvinylidene fluoride membrane. After blocking with 5% BSA in TBST for 2 h, the membrane was incubated overnight with anti-VEGF antibody (ab1316, Abcam) and GAPDH (AF0006, Beyotime) on a shaker at 4 °C and then for 2 h at room temperature with the corresponding secondary antibody. After washing three times with TBST and incubating with enhanced chemiluminescence reagent (SEP100, WSHTBio), the membrane was visualized using achemiluminescent imaging system (Tanon-5200, China).

### Full-thickness diabetic wound model

All procedures on animals were conducted in accordance with the National Institute of Health’s Guidelines for the Care and Use of Laboratory Animals. Male Balb/c mice (20–25 g, 8 weeks) were provided by the Experimental Animal Center of Chongqing Medical University (SCXK2018–0003). All animal experiments were approved by the Animal Ethics Committee of the Chongqing Medical University (Chongqing, China).

These mice were fed a high-fat diet (HFD, MD12033, Jiangsu, China) and given streptozotocin solution (STZ, 50 mg/kg) intraperitoneally for 5 days. After 2 weeks, the blood glucose levels of mice were measured via the tail vein. Mice with blood glucose levels above 16.7 mmol/L on three consecutive occasions were judged to model as type I diabetic mice successfully. Next, the mice were randomly divided into 4 groups (PBS, DCA, Cu-DCA NZs, Cu-DCA NZs + NIR, n = 8). The mice were then anesthetized by the intraperitoneal injection of sodium pentobarbital (0.3% /100 g) to create a circular and full-thickness wound with a diameter of 0.8 cm on the back of the mice. Thereafter, different samples solution were applied to the wound for treatment. The wounds were photographed with a digital camera at 0, 3, 7, 11 and 14 days after the start of treatment, and the images were analyzed by Image J software. The temperature changes of circular wound were monitored using the infrared thermal imager. Also, the body weight and blood glucose of the mice were recorded every two days during the treatment. At the end of the treatment after 14 days, the wound tissue was collected for histological and immunofluorescence analysis.

### *S. aureus*- infected wound model

The *S. aureus* was selected as bacterial strain for mouse infection. Four groups (PBS, DCA, Cu-DCA NZs, Cu-DCA NZs + NIR, n = 3), a circular full-thickness wound was created on the back of the mice as described above. Subsequently, 50 µL of *S. aureus* (10^8^ CFU·mL^− 1^) suspension was added on circular wound to infect all mice. After infection for 24 h, different samples solution were applied to the wound for 3 days. The wound were photographed with a digital camera at 3 days, and agar plate was used to compare the antibacterial activity of each group.

### Histological analysis

The wound tissues were fixed in 4% paraformaldehyde overnight, embedded in paraffin, and serially sectioned at 5 μm. To evaluate the healing process, hematoxylin-eosin (H&E) staining, Masson staining and Picrosirius red staining methods were used. For immunofluorescence analysis, following deparaffinization, antigen retrieval, and blocking, the sections were incubated with primary antibodies. The primary antibodies used were anti-CD31 antibody (ab199012, Abcam), anti-alpha smooth muscle actin antibody (ab7817, Abcam,), anti-TNF alpha antibody (ab183218, Abcam), anti-IL-6 antibody (ab214419, Abcam) and anti-VEGF antibody (ab1316, Abcam). The tissue sections were then washed three times with PBS (pH 7.4) for 5 min each. The sections were then incubated with corresponding secondary antibodies. The nuclei were stained with hematoxylin or DAPI dihydrochloride (C1006, Beyotime), and the slices were observed under an optical microscope. Quantification of the stainings was performed using ImageJ software.

### Statistical analysis

Each experiment was repeated at least three times. The experimental data were statistically analyzed, and the results were expressed as mean ± standard error (SD). One-way analysis of variance (ANOVA) was used for the significant difference analysis of the experimental data. The data were indicated with * *p* < 0.05, ** *p* < 0.01, and *** *p* < 0.001.

### Electronic supplementary material

Below is the link to the electronic supplementary material.

**Additional file 1: Fig S1** Energy Dispersive Spectroscopy mapping (EDS mapping) of Cu-DCA NZs. **Fig S2.** (A) Nitrogen sorption isotherm of Cu-DCA NZs. (B) Pore size distribution of Cu-DCA NZs. **Fig S3.** XRD patterns of Cu-DCA NZs. (Inset is the XRD patterns of Cu-MOF.). **Fig S4.** Thermal stability of Cu-DCA NZs with three circles under NIR irralation (1.0 W cm^− 2^). **Fig S5.** Photothermal conversion efficiency of Cu-DCA NZs under NIR irralation. **Fig S6.** The oxygen saturation produion of H_2_O and DCA solution. **Fig S7.** The oxygen saturation produion of Cu-DCA NZs with different mass ratios. **Fig S8.** The oxygen saturation produion of classical Cu-MOF and Cu-DCA NZs. **Fig S9.** The particle size distribution of Cu- DCA NZs@Ce6 and Cu- DCA NZs@IR808 are measured by DLS. (Inset are the UV–vis absorption spectra and solution photographs of different dyes.). **Fig S10.** The polydisperse index of Cu- DCA NZs after dyes-loaded are measured by DLS. Data are presented as the mean ± SD (n = 3). **Fig S11.** Quantification of Cu- DCA NZs uptake fluorescence intensity with different times. **Fig S12.** The original image of tube formation. **Fig S13.** Complete uncropped western blot analysis images of VEGF expression levels. GAPDH served as an internal reference. (GAPDH: 37 KD, VEGF: 230 KD) **Fig S14.** Photographs of infection wound tissues and agar plates test on day 3 with different treatment. **Fig S15.** Quantify the expression of immunohistochemical VEGF density in different groups on day 14. **Fig S16.** Quantify the expression of immunohistochemical α-SMA density in different groups on day 14. **Fig S17.** Quantify the expression of immunohistochemical CD31 density in different groups on day 14. **Fig S18.** Immunohistochemical staining of IL-6, IL-1β and TNF-α expressed in wounds on day 14. The black arrow indicates the region of positive expression. **Fig S19.** Representative images of H&E staining of the major organs of mice treated with PBS or Cu-DCA NZs + NIR. **Fig S20.** The blood panel analysis results of mice treated with PBS or Cu-DCA NZs + NIR. Data are presented as the mean ± SD (n = 3). **Fig S21.** The blood biochemistry results of mice treated with PBS or Cu-DCA NZs + NIR. Data are presented as the mean ± SD (n = 3). **Table S1**. The concentrations of copper ions of Cu-DCA NZs determined by inductively coupled plasma massspectrometry (ICP-MS)

**Additional file 2:** Video of the reaction of Cu-DCA NZs

**Supplementary Material 3:** Video of the temperature change of Cu-DCA NZs

## Data Availability

All data generated or analyzed during this study are included in this published. article.

## List of abbreviations

DCA: 2,5-dimercaptoterephthalic acid
ROS: Reactive oxygen species
MOF: Metal-organic frameworks SOD:Superoxide dismutase
CAT: Catalase
HUVECs: Human umbilical vein endothelial cells
CCK‑8: Cell counting kit‑8
VEGF: Vascular endothelial growth factor
