# Supplementary material for: Functional molecule-mediated assembled copper nanozymes for diabetic wound healing
Source: J Nanobiotechnology. 2023 Aug 25;21:294. doi: 10.1186/s12951-023-02048-1 (PMC10464099; doi:10.1186/s12951-023-02048-1)
Supplement: Supplementary file 1 — Additional file 1: Fig S1 Energy Dispersive Spectroscopy mapping (EDS mapping) of Cu-DCA NZs. Fig S2. (A) Nitrogen sorption isotherm of Cu-DCA NZs. (B) Pore size distribution of Cu-DCA NZs. Fig S3. XRD patterns of Cu-DCA NZs. (Inset is the XRD patterns of Cu-MOF.). Fig S4. Thermal stability of Cu-DCA NZs with three circles under NIR irralation (1.0 W cm− 2). Fig S5. Photothermal conversion efficiency of Cu-DCA NZs under NIR irralation. Fig S6. The oxygen saturation produion of H2O and DCA solution. Fig S7. The oxygen saturation produion of Cu-DCA NZs with different mass ratios. Fig S8. The oxygen saturation produion of classical Cu-MOF and Cu-DCA NZs. Fig S9. The particle size distribution of Cu- DCA NZs@Ce6 and Cu- DCA NZs@IR808 are measured by DLS. (Inset are the UV–vis absorption spectra and solution photographs of different dyes.). Fig S10. The polydisperse index of Cu- DCA NZs after dyes-loaded are measured by DLS. Data are presented as the mean ± SD (n = 3). Fig S11. Quantification of Cu- DCA NZs uptake fluorescence intensity with different times. Fig S12. The original image of tube formation. Fig S13. Complete uncropped western blot analysis images of VEGF expression levels. GAPDH served as an internal reference. (GAPDH: 37 KD, VEGF: 230 KD) Fig S14. Photographs of infection wound tissues and agar plates test on day 3 with different treatment. Fig S15. Quantify the expression of immunohistochemical VEGF density in different groups on day 14. Fig S16. Quantify the expression of immunohistochemical α-SMA density in different groups on day 14. Fig S17. Quantify the expression of immunohistochemical CD31 density in different groups on day 14. Fig S18. Immunohistochemical staining of IL-6, IL-1β and TNF-α expressed in wounds on day 14. The black arrow indicates the region of positive expression. Fig S19. Representative images of H&E staining of the major organs of mice treated with PBS or Cu-DCA NZs + NIR. Fig S20. The blood panel analysis results of mice treated [file 12951_2023_2048_MOESM1_ESM.docx]

Supporting Information

**Functional molecule-mediated assembled copper nanozymes for diabetic wound healing**

Wenyan Huang^a†^, Ping Xu^a†^, Xiaoxue Fu^a^, Jiaxin Yang^a^, Weihong Jing^a^, Yucen Cai^a^, Yingjuan Zhou^a^, Rui Tao^b^*, Zhangyou Yang^a^*

^a^ College of Pharmacy, Chongqing Key Laboratory for Pharmaceutical Metabolism Research, Chongqing pharmacodynamic evaluation engineering technology research

center, Chongqing 400016, China

^b^ Department of Hepatobiliary Surgery, Bishan hospital of Chongqing Medical University, Bishan Hospital of Chongqing, China

* Corresponding authors.

E-mail address:

yangzhangyou@cqmu.edu.cn (Prof. ZY Yang.)

taorui@vip.126.com (Prof. R Tao.)

^†^ Wenyan Huang and Ping Xu contributed equally to the work.

**Fig S1.** Energy Dispersive Spectroscopy mapping (EDS mapping) of Cu-DCA NZs.


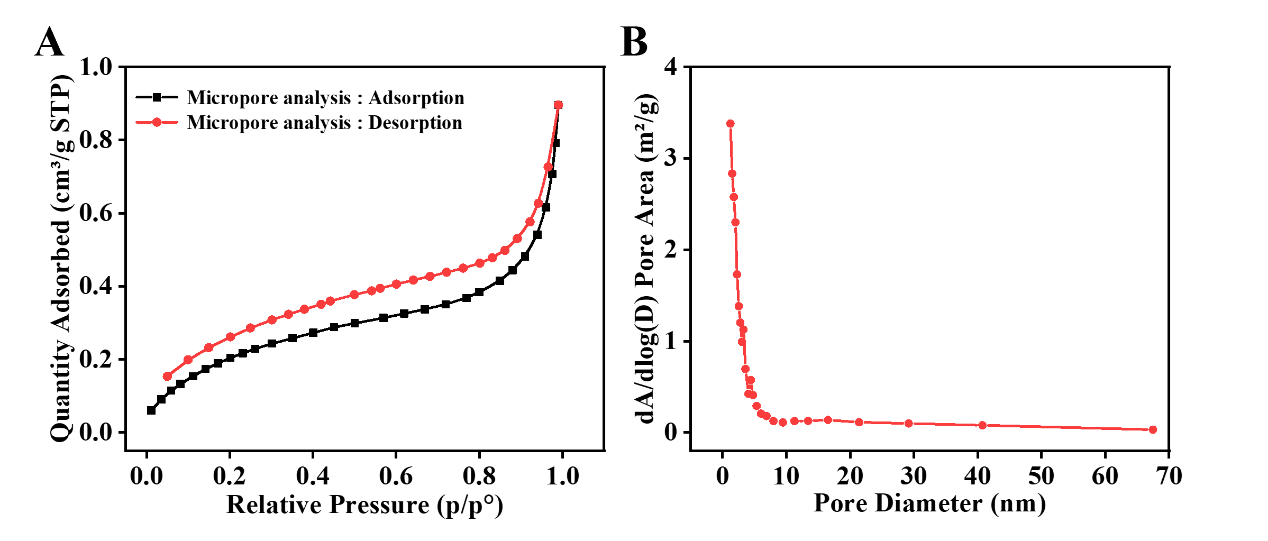


**Fig S2.** A) Nitrogen sorption isotherm of Cu-DCA NZs. B) Pore size distribution of Cu-DCA NZs.

**Fig S3.** XRD patterns of Cu-DCA NZs. (Inset is the XRD patterns of Cu-MOF.).

**Fig S4.** Thermal stability of Cu-DCA NZs with three circles under NIR irralation (1.0 W cm^−2^).

**Fig S5.** Photothermal conversion efficiency of Cu-DCA NZs under NIR irralation.

**Fig S6.** The oxygen saturation produion of H_2_O and DCA solution.

**Fig S7.** The oxygen saturation produion of Cu-DCA NZs with different mass ratios.

**Fig S8.** The oxygen saturation produion of classical Cu-MOF and Cu-DCA NZs.

**
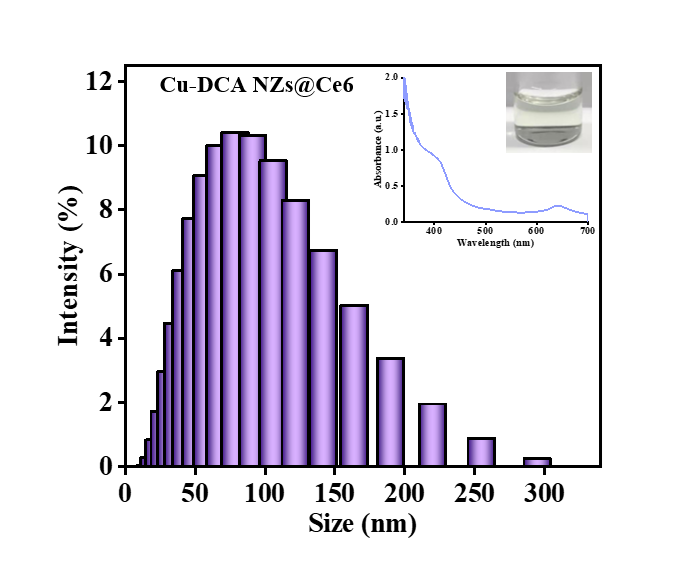
**

**
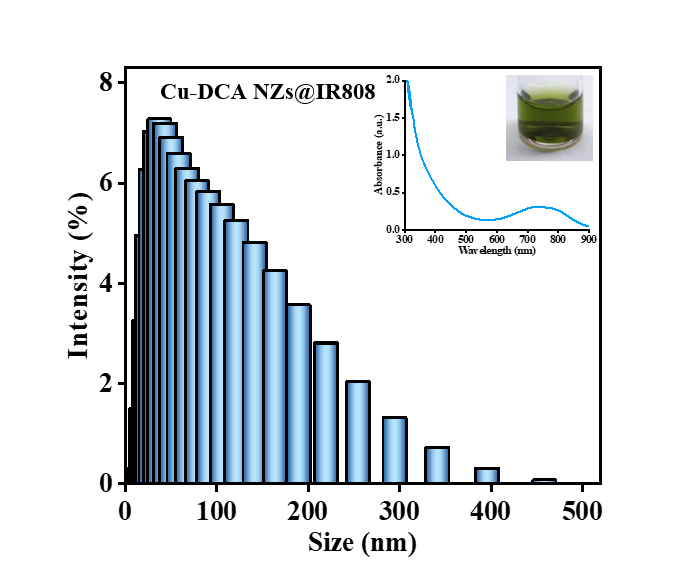
**

**Fig S9.** The particle size distribution of Cu- DCA NZs@Ce6 and Cu- DCA NZs@IR808 are measured by DLS. ( Inset are the UV–vis absorption spectra and solution photographs of different dyes.)

**Fig S10.** The polydisperse index of Cu- DCA NZs after dyes-loaded are measured by DLS. Data are presented as the mean ± SD (n = 3).

**Fig S11.** Quantification of Cu- DCA NZs uptake fluorescence intensity with different times.


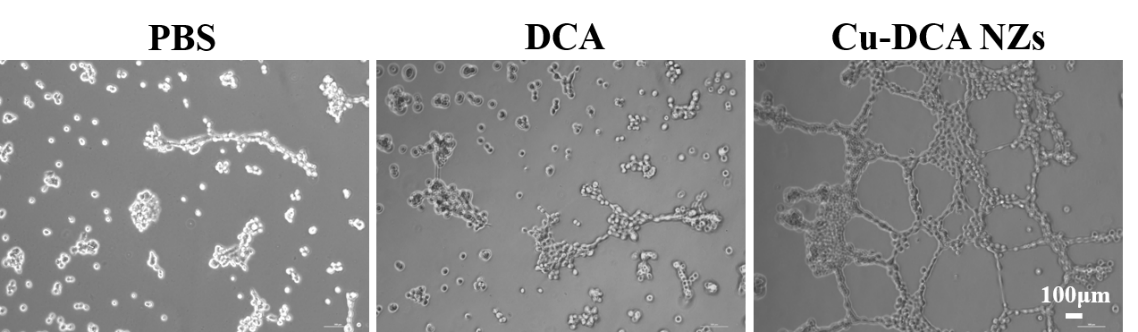


**Fig S12.** The original image of tube formation.


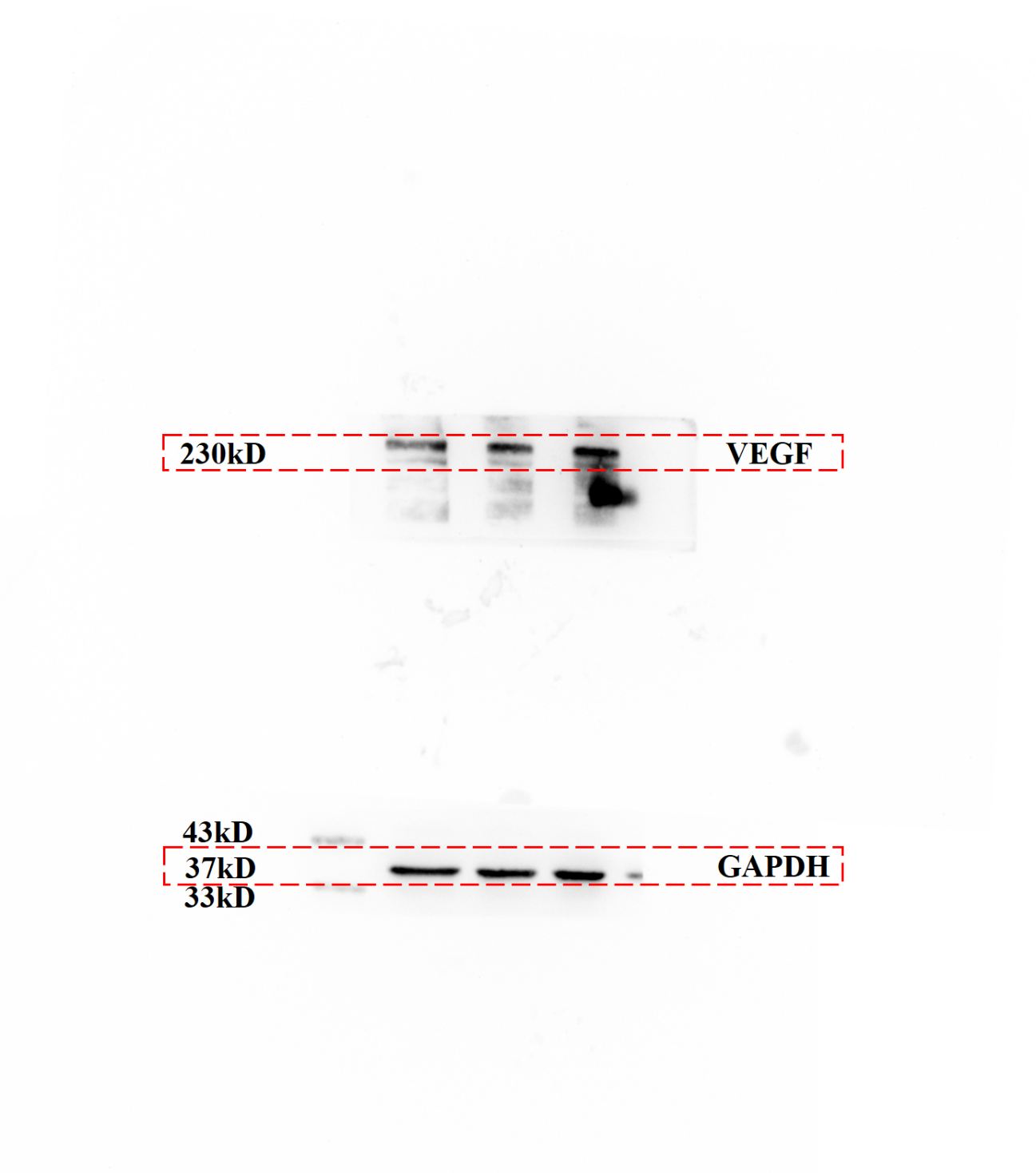


**Fig S13.** Complete uncropped western blot analysis images of VEGF expression levels. GAPDH served as an internal reference. (GAPDH: 37 KD, VEGF: 230 KD)


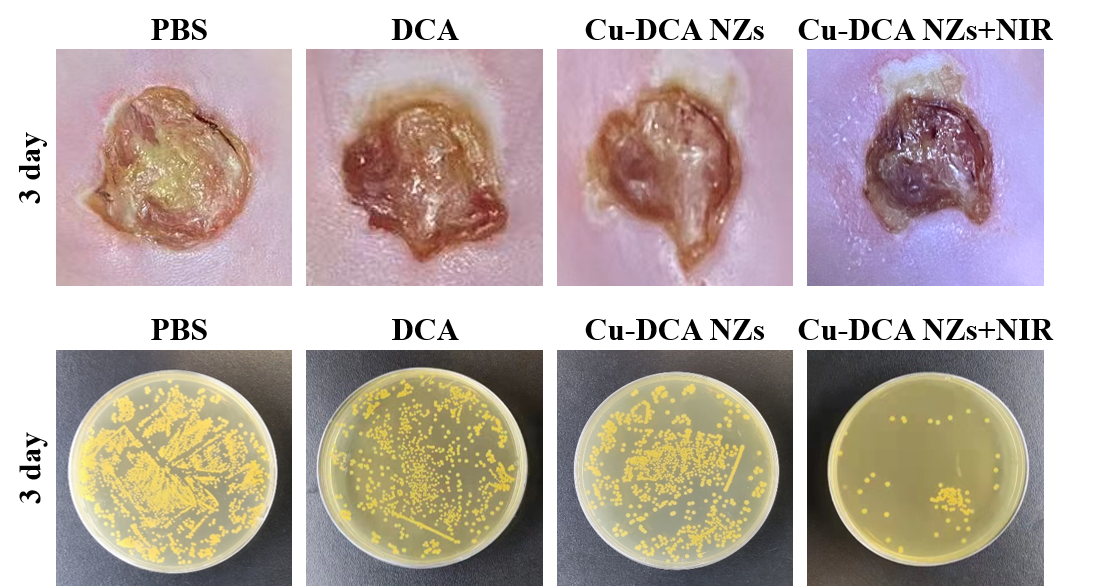


**Fig S14.** Photographs of infection wound tissues and agar plates test on day 3 with different treatment.

**Fig S15.** Quantify the expression of immunohistochemical VEGF density in different groups on day 14. Data are presented as the mean ± SD (n = 3). * *p* < 0.05, ** *p* < 0.01, and *** *p* < 0.001. (Ⅰ: PBS, Ⅱ: DCA, Ⅲ: Cu-DCA NZs, Ⅳ: Cu-DCA NZs + NIR).

**Fig S16.** Quantify the expression of immunohistochemical α-SMA density in different groups on day 14. Data are presented as the mean ± SD (n = 3). * *p* < 0.05, ** *p* < 0.01, and *** *p* < 0.001. (Ⅰ: PBS, Ⅱ: DCA, Ⅲ: Cu-DCA NZs, Ⅳ: Cu-DCA NZs + NIR).

**Fig S17.** Quantify the expression of immunohistochemical CD31 density in different groups on day 14. Data are presented as the mean ± SD (n = 3). * *p* < 0.05, ** *p* < 0.01, and *** *p* < 0.001. (Ⅰ: PBS, Ⅱ: DCA, Ⅲ: Cu-DCA NZs, Ⅳ: Cu-DCA NZs + NIR).


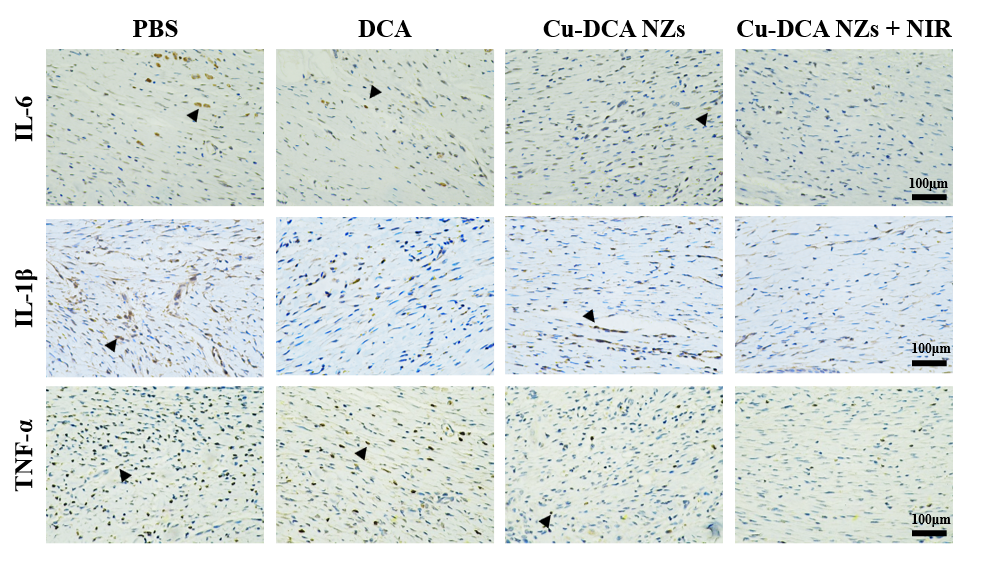


**Fig S18.** Immunohistochemical staining of IL-6, IL-1β and TNF-α expressed in wounds on day 14. The black arrow indicates the region of positive expression.


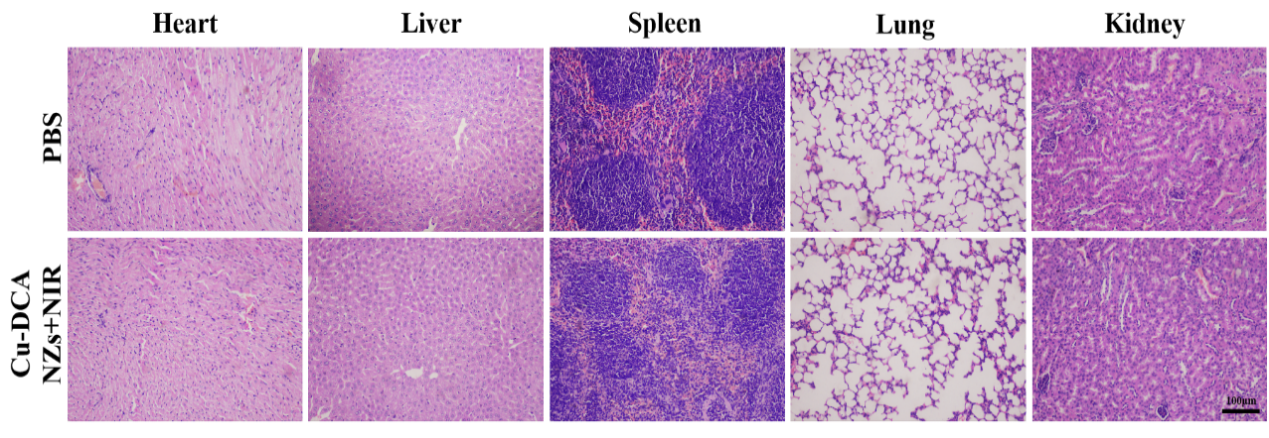


**Fig S19.** Representative images of H&E staining of the major organs of mice treated with PBS or Cu-DCA NZs + NIR.


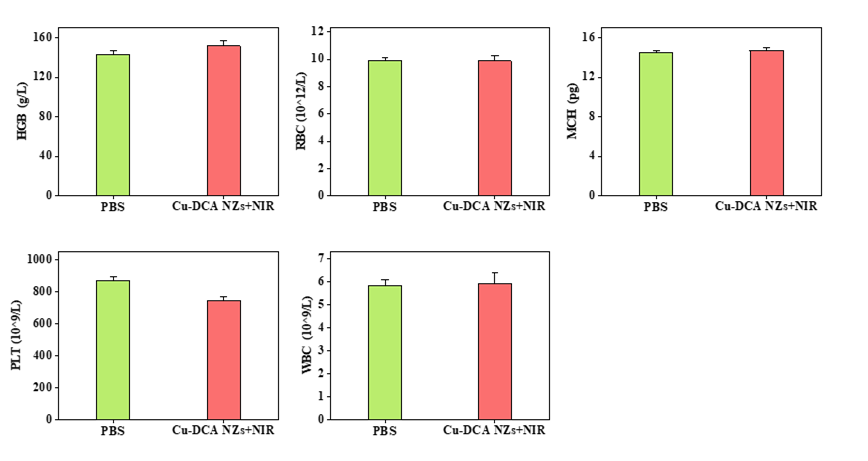


**Fig S20.** The blood panel analysis results of mice treated with PBS or Cu-DCA NZs + NIR. Data are presented as the mean ± SD (n = 3).


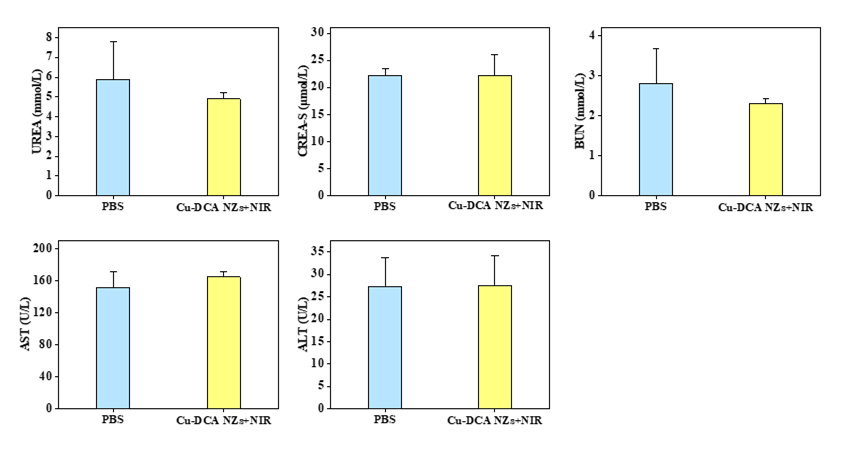


**Fig S21.** The blood biochemistry results of mice treated with PBS or Cu-DCA NZs + NIR. Data are presented as the mean ± SD (n = 3).

**Supporting Tables S1**

**Table S1.** The concentrations of copper ions of Cu-DCA NZs determined by inductively coupled plasma massspectrometry (ICP-MS).

| Concentrations of copper ions | | |
| --- | --- | --- |
|  | 116.304 mg/L= 0.480 mM |  |
